# Identification of TCERG1 as a new genetic modulator of TDP-43 production in *Drosophila*

**DOI:** 10.1186/s40478-018-0639-5

**Published:** 2018-12-12

**Authors:** Marine Pons, Silvia Prieto, Laetitia Miguel, Thierry Frebourg, Dominique Campion, Carles Suñé, Magalie Lecourtois

**Affiliations:** 1 Normandie University, UNIROUEN, Inserm, U1245, IRIB, Rouen, France; 2 Department of Molecular Biology, Institute of Parasitology and Biomedicine “López Neyra” (IPBLN-CSIC), PTS, 18016 Granada, Spain; 3 Department of Genetics, Rouen University Hospital, Rouen, France; 4 Centre Hospitalier du Rouvray, Sotteville-Lès-Rouen, France

**Keywords:** TDP-43, Autoregulation, ALS, FTLD, TCERG1, Drosophila

## Abstract

TAR DNA-binding protein-43 (TDP-43) is a ubiquitously expressed DNA-/RNA-binding protein that has been linked to numerous aspects of the mRNA life cycle. Similar to many RNA-binding proteins, TDP-43 expression is tightly regulated through an autoregulatory negative feedback loop. Cell function and survival depend on the strict control of TDP-43 protein levels. TDP-43 has been identified as the major constituent of ubiquitin-positive inclusions in patients with Amyotrophic Lateral Sclerosis (ALS) and Frontotemporal Lobar Degeneration (FTLD). Several observations argue for a pathogenic role of elevated TDP-43 levels in these disorders. Modulation of the cycle of TDP-43 production might therefore provide a new therapeutic strategy. Using a *Drosophila* model mimicking key features of the TDP-43 autoregulatory feedback loop, we identified *CG42724* as a genetic modulator of TDP-43 production in vivo. We found that CG42724 protein influences qualitatively and quantitatively the *TDP-43* mRNA transcript pattern. CG42724 overexpression promotes the production of transcripts that can be efficiently released into the cytoplasm for protein translation. Importantly, we showed that TCERG1, the human homolog of the *Drosophila* CG42724 protein, also caused an increase of TDP-43 protein steady-state levels in mammalian cells. Therefore, our data suggest the possibility that targeting TCERG1 could be therapeutic in TDP-43 proteinopathies.

**Electronic supplementary material:**

The online version of this article (10.1186/s40478-018-0639-5) contains supplementary material, which is available to authorized users.

## Introduction

In 2006, TAR DNA-binding protein-43 (TDP-43) was identified as the major constituent of ubiquitin-positive inclusions in patients with Amyotrophic Lateral Sclerosis (ALS) and Frontotemporal Lobar Degeneration (FTLD) [2, 51]. In sporadic and familial FTLD/ALS patients, TDP-43 is the most recurrent pathological constituent [70]. TDP-43 proteinopathy can be present in up to 97% of ALS patients, and can be noted in up to 50% of FTLD cases. FTLD-TDP (FTLD with TDP-43 positives inclusions) represents the most frequent FTLD subtypes. Multiple studies identified mutations in the *TARDBP/TDP-43* gene in patients with FTLD/ALS [12, 37, 41, 65, 73], demonstrating that TDP-43 not only represents a pathological hallmark, but also plays a causative role in FTLD/ALS physiopathology. Today, more than 50 missense *TARDBP* mutations have been described [38].

Besides FTLD and ALS, some degree of neuronal TDP-43 pathology has also been reported in a variety of additional neurodegenerative diseases, including Alzheimer’s disease (up to 60% of the patients) [1, 36], corticobasal degeneration (CBD) [72], progressive supranuclear palsy (PSP) [80], Parkinson’s disease [18] and Huntington’s disease [23, 62].

Whatever the disease, pathological TDP-43 manifestations in neurons and glia include the accumulation of insoluble, ubiquitinated and hyperphosphorylated TDP-43 inclusions in the cytoplasm, with a concomitant depletion of TDP-43 from the nucleus [14, 24, 73]. Biochemical analysis of insoluble protein extracts isolated from patient brain tissue also revealed that pathological TDP-43 proteins are partially cleaved to generate carboxy-terminal fragments [2, 51].

TDP-43 is a ubiquitously expressed DNA-/RNA-binding protein [52]. The protein predominantly resides in the nucleus, but is capable of nucleocytoplasmic shuttling [7, 79]. TDP-43 has been linked to numerous aspects of the mRNA life cycle, including transcription, pre-mRNA splicing, mRNA stability, transport, and mRNA translation [22]. TDP-43 also regulates non-coding RNAs (miRNAs, lncRNAs, etc.). Similar to many RNA-binding proteins, TDP-43 expression is tightly regulated through an autoregulatory negative feedback loop. The TDP-43 protein regulates its own protein levels by binding to a sequence called *TDPBR* (for TDP-43 binding region) in the 3’ UTR region of its cognate mRNA [5, 6, 8, 42, 54]. The *TDP-43* pre-mRNA contains multiple alternative introns as well as polyadenylation signals in its last intron (Additional file 1: Figure S1). In steady-state conditions, most TDP-43 production within cells comes from the transcript that uses the optimal polyadenylation site pA1. When TDP-43 concentration rises, increased binding of TDP-43 proteins on the *TDPBR* region interferes with the selection of pA1 and promotes the excision of an alternatively spliced intron (intron 7) containing the pA1 polyadenylation site, and the use of distal suboptimal polyadenylation sites. The resulting isoforms were shown to be retained in the nucleus (thus not available for protein synthesis) or subjected to nonsense-mediated mRNA decay.

Cell function and survival depend on the strict control of TDP-43 protein levels. Numerous studies showed that the perturbation of TDP-43 levels by either increasing or decreasing TDP-43 in animal and cellular models brings severe consequences [17, 32, 44, 61, 71]. Furthermore, several studies have observed an increase in *TDP-43* mRNA and protein levels in various tissues (central nervous system, cerebrospinal fluid, plasma …) of patients suffering from FTLD-TDP or ALS [20, 29, 35, 37, 39, 46, 56, 66, 69, 74, 77]. Interestingly, TDP-43 mutant proteins show various degrees of prolonged half-life and enhanced stability [4, 76], which could lead to an elevated steady-state levels of TDP-43 proteins [9, 64]. Recently, it has been shown in a knock-in mouse model that the FTLD/ALS-linked Q331K mutation perturbs TDP-43 autoregulation, leading to increased TDP-43 expression and gain of function [78]. Altogether, these observations argue for a pathogenic role of elevated TDP-43 levels. Modulation of the TDP-43 production cycle might therefore provide a new therapeutic strategy.

Our group recently developed new *Drosophila* models mimicking key features of the TDP-43 autoregulatory feedback loop, namely alternative splicing events, differential usage of polyadenylation sites, nuclear retention of the transcript and a decrease in steady-state mRNA levels [55]. These transgenic models are based on the expression of an untagged wild-type form of human TDP-43 protein under the control of the *TDPBR* region. Using these animal models, we identified the *Drosophila* gene *CG42724* as a genetic modulator of TDP-43 production in vivo. We showed that *CG42724* overexpression caused a drastic increase of TDP-43 protein steady-state levels, whereas *CG42724* down-regulation resulted in a decrease of TDP-43 accumulation. The study of the underlying molecular mechanisms allowed us to demonstrate that the CG42724 protein influences qualitatively and quantitatively the *TDP-43_TDPBR* mRNA transcripts pattern. CG42724 overexpression promotes the inclusion of the *TDPBR* sensor region and the production of transcripts ending at the pA1 polyadenylation, isoforms that can be efficiently released into the cytoplasm for protein translation. Importantly, we showed that TCERG1, the human homolog of the *Drosophila* CG42724 protein, also caused an increase of TDP-43 protein steady-state levels in mammalian cells.

## Materials and methods

An ethics statement is not required for this work.

### DNA constructs

The fusion construct *GFP::TDP43* was generated using the PCR overlap extension procedure [33]. Each fragments were first generated separately. PCR-amplification of GFP cDNA was achieved with the primers 5’-CCGCTCGAGCGGCAAAATGGTGAGCAAGGGCGAGGAGC-3′ and 5’-CGGTTACCCGAATATATTCAGACTTGTACAGCTCGTCCATGCCG-3′. PCR-amplification of TDP-43 cDNA was achieved with the primers 5’-CGGCATGGACGAGCTGTACAAGTCTGAATATATTCGGGTAACCG-3′ and 5’-TGCTCTAGAGCACTACATTCCCCAGCCAGAAGACTTAGAATCC-3′. Then, these overlapping fragments were both used as template in a PCR reaction, using the primers 5’-CCGCTCGAGCGGCAAAATGGTGAGCAAGGGCGAGGAGC-3′ and 5’-TGCTCTAGAGCACTACATTCCCCAGCCAGAAGACTTAGAATCC-3′. The fusion *GFP::TDP43* PCR product was subcloned in the pcDNA3 vector and sequenced. PCR amplification of the *TDPBR* region was achieved using the *pUAST-TDP-43_TDPBR* plasmid described in [55] and the primers 5’-TGCACTAGTTCACAGGCCGCGTCTTTGACGGTGGG-3′ and 5′- TGCTCTAGAAAAACAAAGACACATATTATTTAAATCAG-3′. The PCR product was then subcloned into the pcDNA3-*GFP::TDP43* vector and sequenced. The pEFBOST7-TCERG1 expression plasmid was previously described in [67]. The expressed TCERG1 protein contains the 11-amino-acid T7 epitope tag at its amino terminus.

### Fly genetics

*Drosophila* were maintained on a 12:12 light/dark cycle on standard cornmeal-yeast agar medium at 25 °C. The following transgenic *Drosophila* strains were used in this study: *UAS-FUS* [19]*, UAS-TDP-43_TDPBR* [55]. The *GMR-Gal4*, *UAS-LacZ*, *UAS-CG42724*^*RNAi*^ (stock #33737 and #55357) lines were obtained from Bloomington Stock Center. Detailed fly genotypes are listed in Additional file 2.

### Cell culture and transfections

HEK293T cells were grown and maintained as previously described [59]. Transfections were performed in 35-mm 6-well plates. Each plate was seeded with approximately 1 × 10^6^ cells 20 h prior to transfection. The cells were grown to approximately 60 to 70% confluence and transfected with the appropriate amounts of the indicated constructs by using the lipofectamine 2000 reagent (Invitrogen, Carlsbad, CA, USA) according to the manufacturer’s protocol. Approximately 48 h after transfection, the cells were harvested and processed for Western blotting analysis.

### Production of the TCERG1 antibody

To express TCERG1 protein in *Escherichia coli*, we amplified two segments containing amino and carboxyl sequences of the TCERG1 cDNA and cloned in frame into the expression vector PGEX2TK (Pharmacia Biotech, Piscataway, NJ, USA). The proteins were expressed as GST fusions under previously published conditions [26]. Purified fusion proteins were used to generate in-house polyclonal antibodies in guinea pigs following standard protocols.

### Mapping of the P{y+}UAS transposon by inverse PCR

Genomic DNA was prepared from 10 flies using the DNeasy Blood and Tissue kit (Qiagen, Hilden, Germany) according to the manufacturer’s instructions. Purified genomic DNA (∼5 μg) was digested by MspI or HinpI (New England Biolabs Inc., Ipswich, MA, USA) for 2–3 h. Digested DNA (∼20.5 μg) was self-ligated (T4 DNA Ligase, New England Biolabs Inc.) overnight at 4 °C in a total volume of 150 μL. To isolate P-element insertion sequence, primer pair OUY31 (*5’ ATTGATTCACTTTAACTTGCAC 3′*) and OUY52 (*5’ ACACAACCTTTCCTCTCAACAA 3′*) was used. The PCR protocol was 95 °C 5 min, 34 cycles of 95 °C 30 s, 55 °C 1 min, 68 °C 2 min, followed by 68 °C 10 min. PCR products were sequenced with OUY31 or OUY52.

### 3’RACE, reverse transcription-quantitative multiplex PCR of short fluorescent fragment (RT–QMPSF) and data calculation

Total RNA extraction and quantification of overall *TDP-43* or *CG42724* mRNA steady-state levels were performed as previously described in [55]. To characterize and quantify the relative abundance of *TDP-43* mRNA splice isoform transcripts, we amplified the 3′ ends of the TDP-43 transcripts using the “3′ RACE System for Rapid Amplification of cDNA Ends” kit (Invitrogen), according to the manufacter’s instructions. Briefly, 500 ng of RNA were converted to cDNA. For qualitative studies (agarose gel electrophoresis), the PCR was performed with 2 μL of the first-strand reaction, 0.2 μM of AUAP primer and 0.2 μM of TDP-43 F3 primer, using the Diamond Taq polymerase (Eurogentec, Liège, Belgium), as recommended by the manufacturer. A touchdown method was used with a DNA Engine (PTC-200) Peltier Thermal Cycler (Bio-Rad Laboratories, Hercules, CA). Cycling times were: 3 min at 95 °C, followed by 40 cycles including (i) denaturation at 95 °C for 10 s, (ii) annealing beginning at 65 °C and ending at 55 °C for 20 s, and (iii) extension at 72 °C for 5 min, with a final extension at 72 °C for 10 min. For quantitative studies, the PCR was performed with 1 μL of the first-strand reaction, 0.08 μM of AUAP primer, 0.08 μM of TDP-43 F3 primer, 0.05 μM of *Cyp1* primers, and 0.06 μM of *RpL13A* primers. Sense primers were 6-FAM-labelled. All primers were used in a single PCR reaction volume of 25 μL. Multiplex fluorescent PCR assays were carried out using 2 mM MgCl2, 1 unit of Diamond Taq polymerase (Eurogentec) and 200 μM of dNTP. After an initial cycle of denaturation at 95 °C for 5 min, 25 cycles were performed consisting of denaturation at 95 °C for 10 s, annealing at 58.8 °C for 30 s, and extension at 72 °C for 1 min 30, and final extension at 72 °C for 10 min, in a DNA engine Peltier Thermal Cycler (Bio-Rad Laboratories). Fluorescent amplicons were separated on an ABI prism 3500 Genetic Analyzer (Applied Biosystems, Foster City, CA, USA), and the resulting fluorescent profiles were analyzed using the GeneMapper 5 software (Applied Biosystems). All QMPSF analyses were performed at least in duplicate. For comparative analyses, the average peak heights obtained for TDP-43 amplicons were compared to the mean peak height obtained for the control amplicons for each genotype. The ratio obtained was set at 100 for the control genotype (*GMR > TDP-43_TDPBR*). TDP-43 expression levels were compared between controls and each of the other genotype by using a Student’s t-test. Primers used in this study are listed in Additional file 3: Table S1.

### Protein extraction and immunoblot analysis

*Drosophila* study: total proteins were prepared by grounding 30 adult fly heads directly in 150 μL Protein Solving Buffer (PSB) (Macherey-Nagel, Düren, Germany), using the TissueLyser LT (Qiagen) through high-speed shaking (50 Hz) of samples in 2 mL microcentrifuge tubes with two 5 mm stainless steel beads for 2 min. Samples were then spun down to collect the lysates, and protein concentrations were measured using the Protein Quantification Assay Kit (Macherey-Nagel).

For sequential extraction of soluble and insoluble proteins, 30 adult fly heads or HEK293T cellular pellets were homogenized in 150 μL Radio Immunoprecipitation Assay (RIPA) buffer (25 mM Tris-HCl pH 7.6, 150 mM NaCl, 1% NP-40, 1% sodium deoxycholate, 0.1% SDS) (Pierce Biotechnology, Rockford, IL, USA), supplemented with a cocktail of protease inhibitors (Sigma-Aldrich) and phosphatase inhibitors (Thermo Fisher Scientific Inc.) using the TissueLyser LT (Qiagen) (two 5 mm stainless steel beads; 50 Hz, 2 min). Samples were then spun down and the lysates transferred to clean tubes. After centrifugation (11,300 x g, 20 min, 4 °C), the supernatant (corresponding to the RIPA-soluble fraction) was reserved in a separate tube while the pellet was washed once in 50 μL of RIPA. The resulting supernatant was pooled with the first one. The remaining pellet was homogenized in 200 μL of urea buffer (urea 9 M, Tris-HCl 50 mM pH 8, CHAPS 1%, and a cocktail of protease and phosphatase inhibitors) and centrifuged at 11,300 x g for 30 min. The supernatant was collected as the urea fraction. Protein concentrations of the soluble fraction were measured using the DC Protein Assay Kit (Bio-Rad Laboratories). Soluble and insoluble proteins were loaded for SDS-PAGE migration in a proportion of 1:1. Proteins were resolved by TGX Stain-Free 12% gels (Bio-Rad Laboratories), and then transferred onto nitrocellulose membrane (Bio-Rad nitrocellulose Turbo transfer packs) for 7 min, 25 V, 2.5 A using the Trans-Blot Turbo system (Bio-Rad Laboratories). Membranes were then blocked using PBS 1x containing 5% non-fat milk and 0.05% Tween, and then incubated with antibodies. Gel loading was normalized by Stain-Free detection of total proteins using a Geldoc™ EZ imager (Bio-Rad Laboratories), as recommended by the manufacturer. The Stain-Free signal obtained in each lane was quantified (ImageLab™ software, Bio-Rad Laboratories). The following primary antibodies were used: rabbit polyclonal anti-TDP-43 (1:5000; Proteintech, Chicago, IL, USA**),** LacZ (1/10,000; Promega, Charbonnières-les-Bains, France), FUS (1/5000; Bethyl Laboratories, Inc. Montgomery, TX, USA), TCERG1 (1:5000). Membranes were incubated with secondary peroxidase-labelled anti-mouse, anti-guinea or anti-rabbit antibodies (1:10,000) from Jackson Immunoresearch Laboratories (WestGrove, PA, USA), and signals were detected with chemiluminescence reagents (ECL Clarity, Bio-Rad Laboratories). Signals were acquired with a GBOX (Syngene, Cambridge, UK), monitored by the Gene Snap software (Syngene). The signal intensity in each lane was quantified using the Genetools software (Syngene), and normalized with the Stain-Free signal quantified in the corresponding lane.

### RNA and protein subcellular fractionation

Fifty newly-eclosed adult fly heads were ground to powder using the TissueLyser LT (Qiagen) through three one-minute cycles of high-speed shaking (50 Hz) in 1.5 mL microcentrifuge tubes with two 2.5 mm stainless steel beads. Samples were then gently homogenized in 240 μL of fractionation buffer (Hepes 10 mM, NaCl 10 mM, MgCl2 3 mM, NP-40 0.5%, RNAse inhibitor 100 u/mL (Promega, Fitchburg, WI, USA)) on ice and centrifuged at 100 x g for 30 s to spin down debris. Lysates were then centrifuged at 2300 x g for 5 min at 4 °C to separate nuclei from cytoplasm. Nuclei (pellet) were washed 3 times in 500 μL of fractionation buffer and stored overnight at − 80 °C. 20 μl of Sodium acetate 3 M pH 5.2 and 600 μL of Ethanol 100% were added to cytoplasmic fractions (Supernatant). Samples were vortexed vigorously and then stored at − 80 °C overnight. Cytoplasmic proteins and nucleic acids were then pelleted at 14,000 x g for 15 min at 4 °C and washed once with 500 μL of Ethanol 70%. Proteins and RNA derived from nuclear and cytoplasmic fractions were then extracted using the Nucleospin RNA/protein kit (Macherey-Nagel) using the manufacturer’s recommendations.

### Statistical analysis

All n reported are biological replicates. All statistical analyses were performed using a two-tailed Student’s t-test with Welch’s correction for unequal variances (GraphPad, San Diego, CA, USA). Data on graphs are expressed as mean values, error bars representing the standard error of the mean (SEM). For significance symbols, one asterisk means *p* < 0.05, two asterisks mean *p* < 0.01, and three asterisks mean *p* < 0.001.

## Results

### Identification of *CG42724* as a modulator of TDP-43 production

**Fig. 1:**
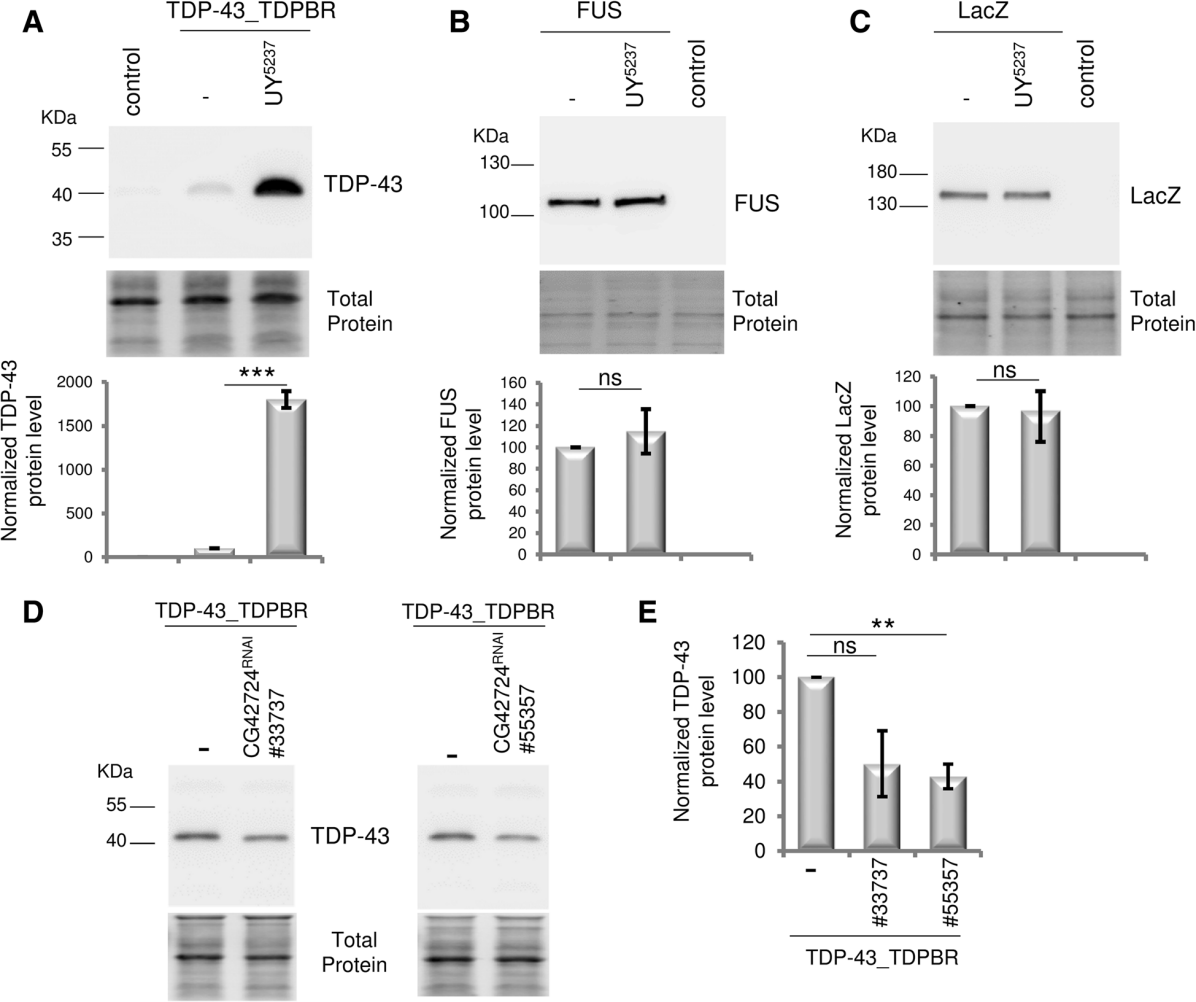
*CG42724* expression modulates TDP-43 production. **a-c** Western Blot analyses of proteins extracted from transgenic flies expressing *UAS-TDP-43_TDPBR* (**a**), *UAS-FUS* (**b**) or *UAS-LacZ* (**c**) constructs, in the presence or the absence of the *P(UY)5237* element, under the control of the *GMR-Gal4* driver line. Control flies: *GMR-Gal4 > +*. Expression of TDP-43, FUS and LacZ proteins was qualified using specific antibodies. Representative blots are shown (*n* ≥ 4). Total protein was used as loading control and the normalized expression of the proteins of interest is reported in the graphs (mean ± SEM). Genotypes *GMR-Gal4 > UAS-TDP-43_TDPBR*, *GMR-Gal4 > UAS-FUS* and *GMR-Gal4 > UAS-LacZ* were arbitrarily set at 100 arbitrary units. The *P(UY)5237* element caused a drastic increase of TDP-43 protein steady-state levels (*n* = 8, *p* = 0.0001), but did not modify significantly FUS (*n* = 4, *p* = 0.524) or LacZ (n = 4, *p* = 0.822) expression. **d, e** Western Blot analyses of total proteins extracted from transgenic flies expressing *UAS-TDP-43_TDPBR* with or without RNAi constructs targeting *CG42724* (#33737 or #55357) under the control of the *GMR-Gal4* driver line (**d**). Blots were probed with an anti-TDP-43 antibody and representative blots are presented (n = 4). Total protein was used as the loading control. The normalized expression of the TDP-43 protein is reported in the graphs (mean ± SEM) (**e**). Control (*GMR-Gal4 > UAS-TDP-43_TDPBR)* was arbitrarily set at 100 arbitrary units. The reduction of *CG42724* expression decreased TDP-43 production by about 50% (CG42724^RNAi^#33737: *n* = 4, *p* = 0.0659, CG42724^RNAi^#55357: n = 4, *p* = 0.0040). **a-e** Protein levels were compared between both genotypes by using Student’s t-test. ns: not significant, **: *p* < 0.01. ***: *p* < 0.001

In the course of a P[UAS]-based misexpression screen for modifiers of TDP-43 production, we screened part of the UY collection [47]. For the screen, we crossed *GMR-Gal4 > TDP-43_TDPBR* females to UYi males and assessed the F1 progeny for TDP-43 production by western blot analysis. The *GMR-Gal4* driver line is expressed in all cells of the developing and adult eyes, including the photoreceptor neurons as well as accessory pigment cells. We identified a *P[UAS]*-insertion line (*UY5237*) that significantly increased TDP-43 production (Fig. 1a). As expected western blot analysis of total protein extracts from newly-eclosed adult heads revealed a single band with an apparent molecular mass of ~ 43 kDa that corresponded to the predicted size of the 414 amino acid TDP-43 sequence. No signal was detected in control flies *(GMR > +)*, indicating that the human TDP-43 antibody did not recognize *Drosophila* proteins, including the *Drosophila* homolog TBPH. Normalization of the amount of TDP-43 proteins using the stain-free technology [58] showed that the *P(UY)5237* element caused a drastic increase (~ 18-fold) of TDP-43 protein steady-state levels (*p* = 0.0001). In contrast, the *P(UY)5237* element did not modify significantly FUS (*p* = 0.524) (Fig. 1b) and LacZ (*p* = 0.822) (Fig. 1c) expression.

**Fig. 2:**
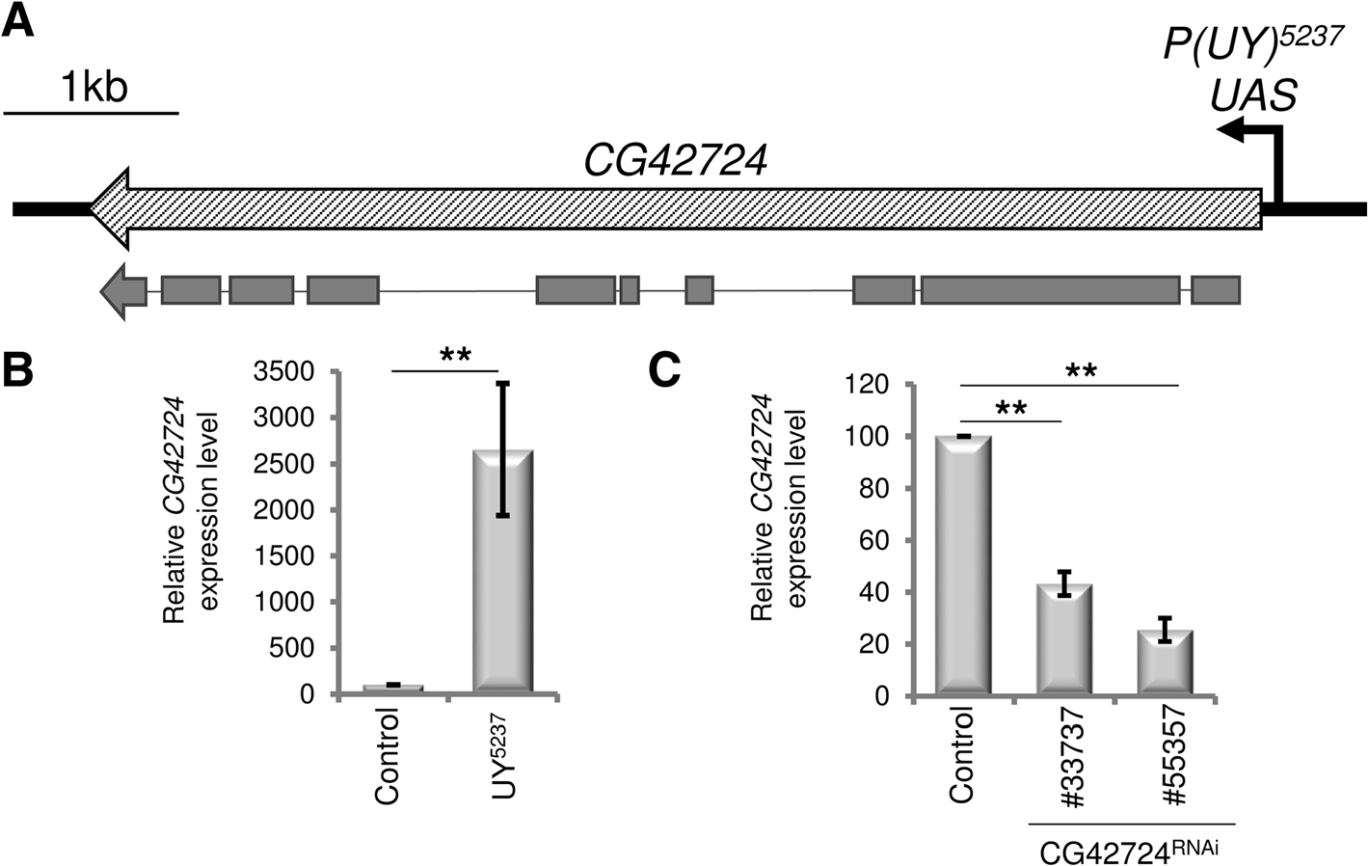
Characterization of the transposon *UY5237* line. **a** The *CG42724* transcription unit is represented by the filled rectangle. Exons are represented by rectangles below the transcription unit, and introns as a line. The arrow represents the orientation of transcription from the *P{y +}UAS* transposon in the *UY5237* transgenic line. Scale bar (upper right) is 1000 bp. Schematic representation adapted from FlyBase. (**b**, **c**) Quantification of the *CG42724* mRNA steady-state levels by RT-QMPSF experiments. Total RNA were extracted from *GMR-Gal4 > +* (control), *GMR-Gal4 > UY5237*, *GMR-Gal4 > UAS-CG42724*^*RNAi*^ *#33737* or *GMR-Gal4 > UAS-CG42724*^*RNAi*^ *#55357* transgenic flies. The graph represents mean ± SEM after normalization with *Cyp1* (reference gene). Controls were arbitrarily set at 100 arbitrary units. The mRNA levels were compared between both genotypes by using Student’s t-test. **: *p* < 0.01. **b** *CG42724* mRNA expression in flies heterozygous for the *UY5237* transposon was significantly increased, compared to control flies (n = 8, *p* = 0.009). **c** Expression of RNAi constructs targeting *CG42724* (#33737 or #55357) significantly reduced *CG42724* mRNA steady-state levels, relative to control flies (*CG42724*^*RNAi*^*#33737* n = 4, *p* = 0.0011, *CG42724*^*RNAi*^*#55357 n* = 3, *p* = 0.0037)

PCR rescue experiments were then performed to identify the insertion point and orientation of the transposon by comparing the sequence of the flanking genomic DNA to the *Drosophila* genome sequence database. The *UY5237* line corresponds to a *P{y+}UAS* transposon inserted 170 bp upstream of the *CG42724* gene, potentially driving transcription of the gene in a Gal4-dependent manner (Fig. 2a). To validate that the transposon insertion in the *UY5237* line leads to upregulation of the related downstream gene in the presence of Gal4, we performed an RT-QMPSF (reverse transcription-quantitative multiplex PCR of short fluorescent fragments). This assay is based on simultaneous PCR amplification of short fluorescent fragments and allows the comparative quantitative analysis of mRNA. We compared the levels of *CG42724* transcripts in *GMR-Gal4 > +* and *GMR-Gal4 > UY5237 Drosophila* heads. We indeed observed a significant upregulation of the *CG42724* transcripts (about 27 fold, *p* = 0.009) in *GMR-Gal4 > UY5237* flies, compared to control flies (*GMR-Gal4 > +*) (Fig. 2b, Additional file 4: Figure S2).

To confirm the role of *CG42724* on TDP-43 proteins steady-state levels, we then tested two independent *CG42724*^*RNAi*^ *Drosophila* transgenic lines that target two different regions of the *CG42724* mRNA (Additional file 4: Figure S2A)*.* First, we validated the efficiency of both RNAi by assessing the RNAi-mediated decrease of *CG42724* expression by RT-QMPSF. We found that expression of RNAi constructs targeting *CG42724* significantly reduced *CG42724* mRNA steady-state levels (*CG42724*^*RNAi#33737*^ *p* = 0.0011, *CG42724*^*RNAi#55357*^ *p* = 0.0037) (Fig. 2c). Then, we quantified TDP-43 proteins steady-state levels and we found that the reduction of *CG42724* expression decreased TDP-43 production by about 50% (*CG42724*^*RNAi#33737*^, *p* = 0.0659, *CG42724*^*RNAi#55357*^, *p* = 0.0040) (Fig. 1d,e). The opposing effects observed on TDP-43 production from RNAi and overexpression approaches strongly argue that *CG42724* acts as a genetic modulator of TDP-43 production.

### Effect of the CG42724-mediated regulation of TDP-43 production on TDP-43 solubility and TDP-43 phenotypic severity

**Fig. 3:**
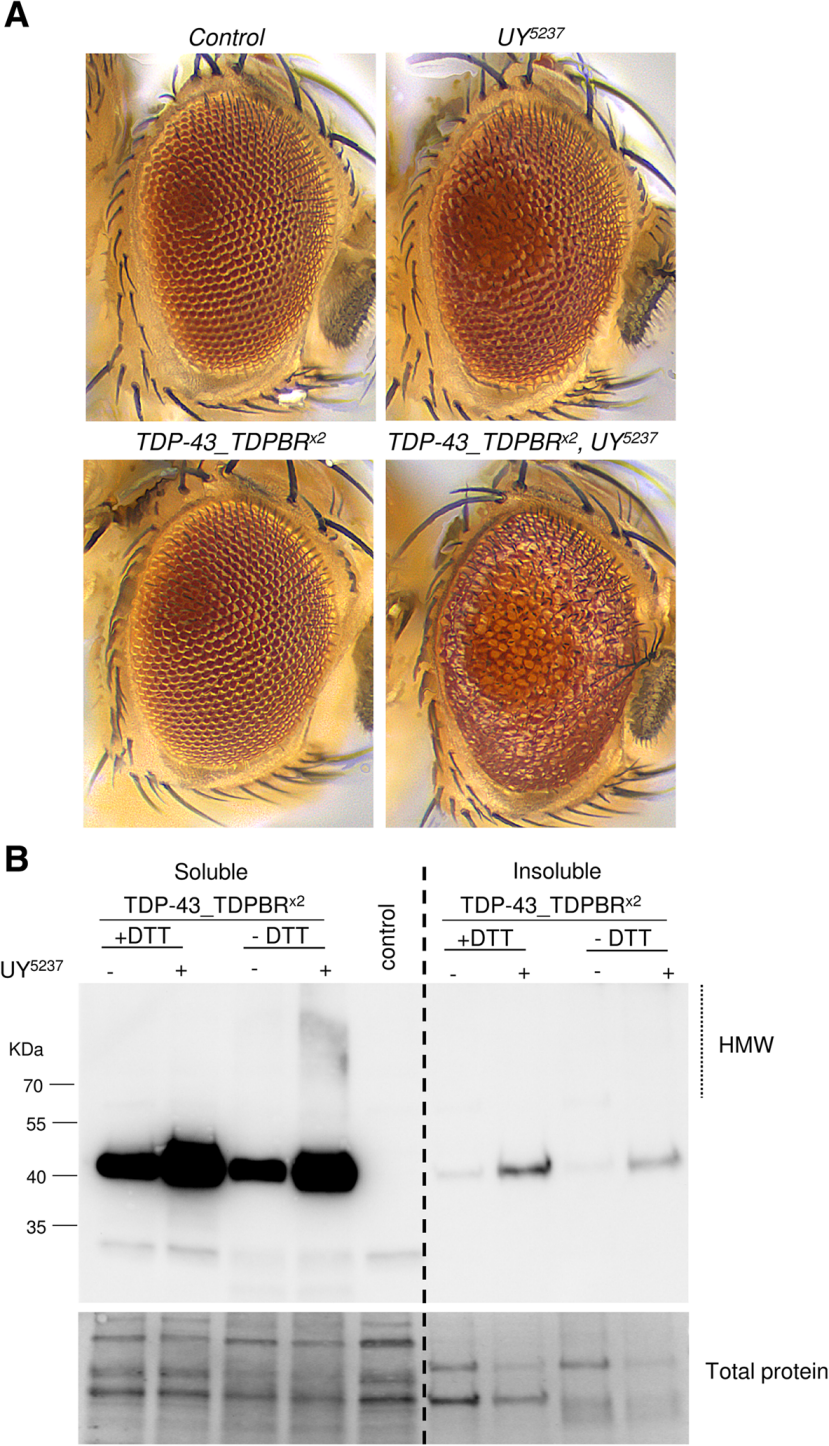
CG42724-mediated increase of TDP-43 production results in the appearance of insoluble TDP-43 aggregates and causes cellular toxicity in *Drosophila* retina. **a** Light micrographs of newborn *Drosophila* adult eyes raised at 23 °C. Compared to control flies (*GMR-Gal4*^*x2*^ > +), *TDP-43_TDPBR*^*x2*^ (*GMR-Gal4*^*x2*^ *> UAS-TDP-43_TDPBR*^*x2*^) expression alone triggered no structural defects. Flies overexpressing *CG42724* (*GMR-Gal4* ^*x2*^ *> UY5237*) displayed alteration of the external eye aspect (“rough-eye phenotype”). Coexpression of *CG42724* and *TDP-43_TDPBR*^*x2*^ (*GMR-Gal4* ^*x2*^ *> UAS-TDP-43_TDPBR*^*x2*^*, UY5237*) enhanced the severity of the “rough-eye phenotype” in a synergistic manner. **b** Western blot analyses of TDP-43 proteins extracted from flies expressing *TDP-43_TDPBR*^*x2*^ with or without the *P(UY)5237* transposon under the control of the *GMR-Gal4* driver, and control flies bearing only the *GMR-Gal4* transgene. Proteins were sequentially extracted in RIPA (soluble) and Urea (insoluble) buffers. Samples were loaded with (+ DTT) or without (− DTT) reducing agent. Blots were probed with an anti-TDP-43 antibody and representative blots are presented (n = 4). Total protein was used as the loading control. *CG42724*-mediated increased expression of TDP-43 resulted in appearance of DTT-sensitive high molecular weight (HMW) species

We next assessed whether CG42724-mediated increased expression of TDP-43 resulted in cellular toxicity in *Drosophila* retina. We observed that flies carrying a single copy of the *TDP-43_TDPBR* construct with the *P(UY)5237* element displayed no obvious external phenotype, compared to control flies (Additional file 5: Figure S3). However, when we maximized TDP-43 protein expression by making use of flies bearing two copies each of the *TDP-43_TDPBR* transgene, we found that *CG42724* co-expression caused strong synergistic effects (Fig. 3a). Compared to control eyes, *TDP-43_TDPBR*^*x2*^ expression induced no discernible phenotype. In contrast, *CG42724* overexpression resulted in a rough-eye phenotype. Co-expression of *TDP-43_TDPBR*^*x2*^ and *CG42724* in the eye was associated with a more severely disorganized rough-eye phenotype.

Interestingly, we also observed that *CG42724* overexpression is associated with the appearance of TDP-43 high-molecular weight (HMW) species (Fig. 3b). Adult heads from *GMR > TDP-43_TDPBR*^*x2*^ or *GMR > TDP-43_TDPBR*^*x2*^*, UY5237* transgenic flies were extracted with RIPA buffer followed by extraction in urea buffer to recover insoluble TDP-43. Samples were loaded with or without reducing agent (−DTT) to prevent dissociation of putative HMW forms. Only a faint signal was detected in the insoluble urea fraction, indicating that TDP-43 species were mainly recovered as soluble forms in *Drosophila*. As expected, the expression of *CG42724* increased the TDP-43 protein steady-state levels. TDP-43 proteins were detected as either full-length monomeric forms or HMW species, with TDP-43 proteins being the most prone to form aggregates in the presence of the *UY5237* transgene. When samples were analysed in the presence of the reducing agent (+DTT), we observed a decrease of the HMW forms and, concomitantly, an increase of TDP-43 monomeric species, indicating that these complexes were indeed DTT-sensitive. Altogether, these data showed that CG42724-mediated increased expression of TDP-43 results in the appearance of TDP-43 HMW species and is associated with cellular toxicity in *Drosophila* retina.

### CG42724-mediated regulation of TDP-43 production depends mainly on the presence of the *TDPBR* region

**Fig. 4:**
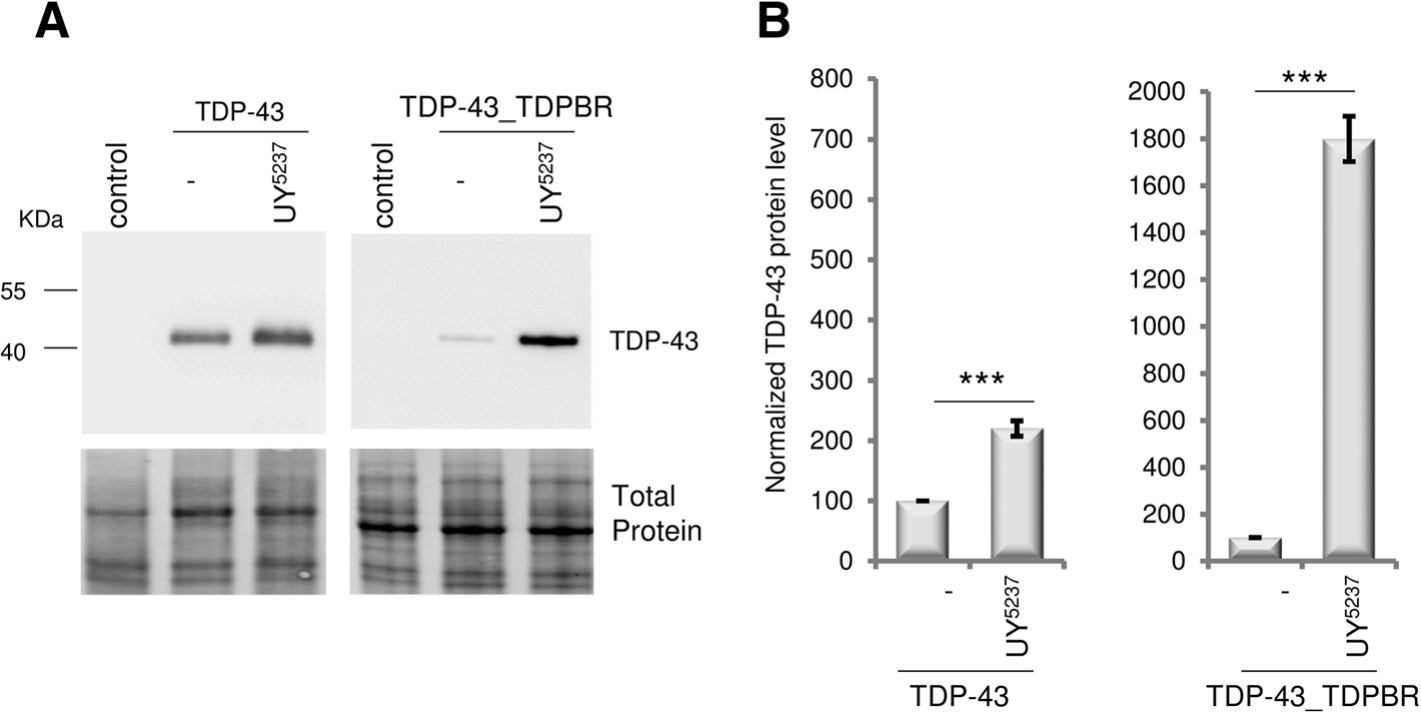
CG42724-mediated regulation of TDP-43 production depends mainly on the presence of the *TDPBR* region. Western Blot analyses of proteins extracted from transgenic flies expressing the *UAS-TDP-43_TDPBR* or the *UAS-TDP-43* constructs, in the presence or the absence of the *P(UY)5237* element, under the control of the *GMR-Gal4* driver line. Control flies: *GMR-Gal4 > +*. **a** Blots were probed with an anti-TDP-43 antibody and representative blots are presented (*n* ≥ 7). Total protein was used as the loading control. **b** The normalized expression of the TDP-43 protein is reported in the graphs (mean ± SEM). Genotypes *GMR-Gal4 > UAS-TDP-43_TDPBR* and *GMR-Gal4 > UAS-TDP-43* were arbitrarily set at 100 arbitrary units. Protein levels were compared between both genotypes by using Student’s t-test. ***: p < 0.001. The *P(UY)5237* element caused a drastic increase of TDP-43 protein steady-state levels in the context of the *UAS-TDP-43_TDPBR* construct (n = 8, p = 0.0001), but only a slight rise when the *UAS-TDP-43* construct was expressed (*n* = 7, *p* = 0.0002)

To address the molecular mechanisms underlying these genetic interactions, we first determined whether the *TDPBR* region contributes to the CG42724-mediated regulation of TDP-43 protein production using the *UAS-TDP-43* construct (no *TDPBR* region) previously described in [55]. Western blot analysis of total protein extracts showed that, in contrast to what we observed with the *UAS-TDP-43_TDPBR* construct (about 18 fold, *p* = 0.0001), overexpression of *CG42724* caused only a 2-fold increase in TDP-43 protein steady-state levels (*p* = 0.0002) (Fig. 4), demonstrating that CG42724-mediated regulation of TDP-43 protein production predominantly depends on the presence of the *TDPBR* region.

### CG42724 expression regulates TDP-43 production by regulating alternative splicing events and nucleocytoplasmic export of *TDP-43* mRNAs

We previously demonstrated that the self-regulatory process of TDP-43 protein steady-state levels in flies depends on alternative splicing events, differential usage of polyadenylation sites, nuclear retention of the transcript and a decrease in steady-state mRNA levels [55]. Interestingly, the *CG42724* gene encodes a homolog of the human *TCERG1 (Transcription elongation regulator 1)* gene (Additional file 6: Figure S4). Human TCERG1 is a nuclear protein that has been implicated in transcription and pre-mRNA-splicing regulation. TCERG1 physically couples transcription elongation and splicing events by interacting with splicing factors and the RNA polymerase II. We therefore sought to determine which of the cellular processes involved in the TDP-43 autoregulatory feedback loop were affected by *CG42724* overexpression.

**Fig. 5:**
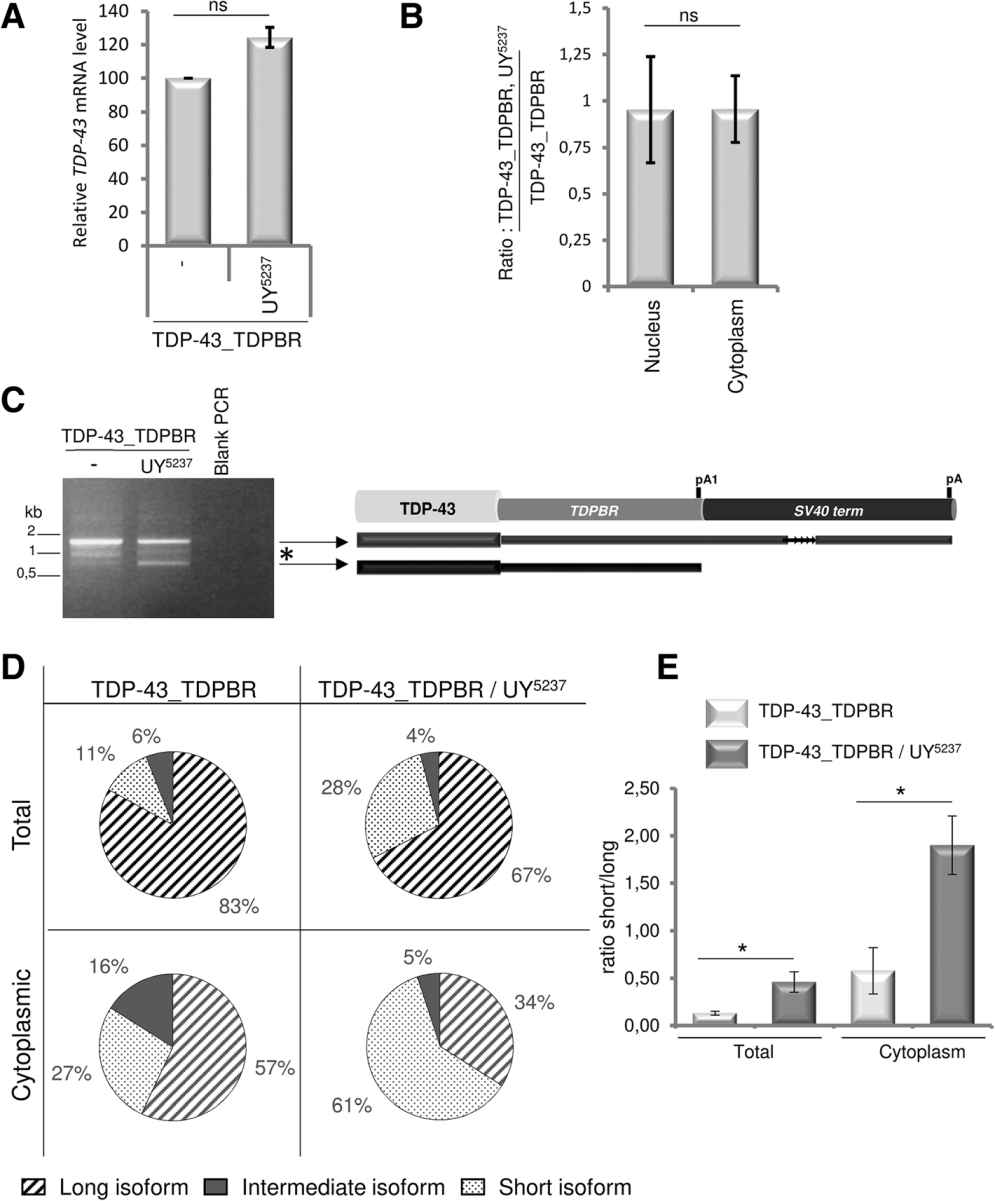
CG42724 influences TDP-43 production by regulating qualitatively and quantitatively the pattern and the nucleocytoplasmic export of *TDP-43* mRNA transcripts. **a** Quantification of the *TDP-43* mRNA steady-state levels by RT-QMPSF experiments in *GMR-Gal4 > UAS-TDP-43_TDPBR* and *GMR-Gal4 > UAS-TDP-43_TDPBR, UY5237* flies. The graph represents mean ± SEM after normalization with *Cyp1* (reference gene). Controls were arbitrarily set at 100 arbitrary units. The mRNA levels were compared between both genotypes by using Student’s t-test. ns: not significant. *CG42724* expression did not significantly influence *TDP-43* mRNA steady-state levels (n = 4, *p* = 0.055). **b** Total RNA from cytoplasmic and nuclear fractions were isolated from *GMR > TDP-43_TDPBR* or *GMR > TDP-43_TDPBR, UY5237* transgenic flies. The graph represents mean ± SEM of *TDP-43* mRNA levels detected by RT-QMPSF experiments, after normalization with *Cyp1.* Controls were arbitrarily set at 100 arbitrary units. *TDP-43* expression levels were compared by using Student’s t–test. ns: not significant. *CG42724* expression did not modulate global *TDP-43* mRNA nucleocytoplasmic export (n = 3, *p* = 0.992). **c** Agarose gel electrophoresis of the PCR products in the 3’ RACE analysis. The 3’ RACE experiments were performed using RNA described in (**a**). Left: representative gel image (*n* > 10). Expression of *CG42724* resulted in a qualitative distinct pattern. Right: schematic representation of the long and the short *TDP-43_TDPBR* mRNA variants detected in flies. **d** Quantification of the relative abundance of the *TDP-43_TDPBR* mRNA variants by 3′ RACE PCR amplification combined to QMPSF methodology, in *GMR-Gal4 > UAS-TDP-43_TDPBR* and *GMR > TDP-43_TDPBR, UY5237* transgenic flies. The mRNA fractions from the cytoplasm and from whole cells (total RNA) were analyzed. Three fluorescent peaks corresponding to a short isoform, intermediate species and a long isoform were detected after separation by capillary electrophoresis (Additional file 7: Figure S5C). **e** The graph represents the ratio of the relative abundance of the short isoform/long isoform in each experimental condition presented in (**d**) (n = 4, *GMR-Gal4 > UAS-TDP-43_TDPBR*: *p* = 0.038, *GMR > TDP-43_TDPBR, UY5237: p* = 0.017)

We first evaluated whether changes in *TDP-43* steady-state mRNA levels could account for the observed modulation at the protein level. We performed RT-QMPSF experiments on *GMR > TDP-43_TDPBR* or *GMR > TDP-43_TDPBR, UY5237* transgenic flies, using pairs of primers that can detect all isoforms of the *TDP-43* mRNA (F1/R1 and F2/R2, Additional file 7: Figure S5A and Additional file 3: Table S1). As shown in Fig. 5a and Additional file 7: Figure S5B, modulation of *CG42724* expression resulted in a slight, but not significant statistical increase of overall *TDP-43* mRNA levels compared to the control (*p* = 0.055).

We next asked whether *CG42724* expression could affect the nucleocytoplasmic export of *TDP-43* mRNAs. We performed cell fractionation (Additional file 8: Figure S6), and extracted total RNAs from nuclear and cytoplasmic fractions. Quantification of *TDP-43* steady-state mRNA levels was achieved again by RT-QMPSF, using the F1/R1 and F2/R2 primers (Additional file 3: Table S1 and Additional file 7: Figure S5A). If *CG42724* expression modulates *TDP-43* mRNA nucleocytoplasmic export, we expect to have different *TDP-43_TDPBR, UY5237 / TDP-43_TDPBR* ratios in the nuclear and the cytoplasmic compartments. However, we detected similar ratio in both compartments (*p* = 0.992) (Fig. 5b), indicating that *CG42724* expression did not modulate *TDP-43* mRNA nucleocytoplasmic export.

We also examined whether the regulation of TDP-43 production by *CG42724* occured via changes in alternative splicing events and/or differential usage of polyadenylation sites, performing 3’ Rapid Amplification of cDNA Ends (RACE) experiments. PCR products were amplified using a human TDP-43-specific primer (F3, Additional file 7: Figure S5A and Additional file 3: Table S1) and an oligo-dT adapter primer. Agarose gel electrophoresis revealed, as previously described in [55], that *TDP-43_TDPBR* transgenic flies displayed a complex pattern of 3′ RACE PCR amplification (Fig. 5c), with a ~ 1.2 kb predominant band and lower migrating species. Co-expression of *CG42724* resulted in a qualitative distinct pattern. Two main bands were now observed: a ~ 1.2 kb fragment (long isoform) and a smaller one of ~ 800 bp (short isoform). The sequencing of these two major bands revealed that they corresponded to alternative transcripts of different sizes resulting from differential usage of polyadenylation sites. Note that we failed to characterize the intermediate band by sequencing (asterisk, Fig. 5c). These species could correspond to heteroduplexes. To quantify the relative abundance of these spliced isoforms, we combined 3′ RACE PCR amplification with QMPSF technology. In accordance with what we observed on agarose gel (Fig. 5c), we detected three fluorescent peaks corresponding to the expected amplicon sizes (short isoform: ~ 840 bp, intermediates species: ~ 1000 bp, long isoform: ~ 1225 bp) (Additional file 7: Figure S5C). Accurate quantification of the relative amount of spliced isoforms showed that *CG42724* expression resulted in an increased relative amount of the short isoform (from 10.71 +/− 1.7% to 28.26 +/− 5.17%, *n* = 6, *p* = 0.0177), with a concomitant decrease of the long isoform (from 83.08 + /− 1.32% to 67.47 +/− 4.79%, n = 6, *p* = 0.0212) (Fig. 5d, upper 2D-pies). Altogether, these data revealed that *CG42724* expression affected qualitatively and quantitatively the *TDP-43_TDPBR* mRNA transcripts pattern.

Because alternative transcripts could display distinct nucleocytoplasmic export efficiency, we then achieved 3′ RACE PCR amplification combined with QMPSF experiments after nucleocytoplasmic fractionation. If both *TDP-43* mRNA isoforms were similarly distributed between the nuclear and cytoplasmic fractions, we would expect similar short/long (S/L) isoforms ratios of *TDP-43* mRNA levels, whatever the experiments were performed using total mRNA or mRNA extracted from the cytoplasmic compartment. Quantification of the relative abundance of splice isoforms showed that the S/L ratio in the cytoplasmic compartment was significantly higher compared to that quantified in total mRNA (*GMR > TDP-43_TDPBR* flies: ~ 2.7 fold-change, *GMR > TDP-43_TDPBR, UY5237*: ~ 3.3 fold-change) (Fig. 5d, lower 2D-pies, E), demonstrating that short *TDP-43* mRNA isoforms were more prone to be exported to the cytoplasm than long isoforms.

Thus, in our experimental system, we found that CG42724 overexpression promotes the production of transcripts including *TDPBR* sensor region and ending at the pA1 polyadenylation site, isoforms that can be efficiently released into the cytoplasm.

### Human TCERG1 controls TDP-43 production in mammalian cells

**Fig. 6:**
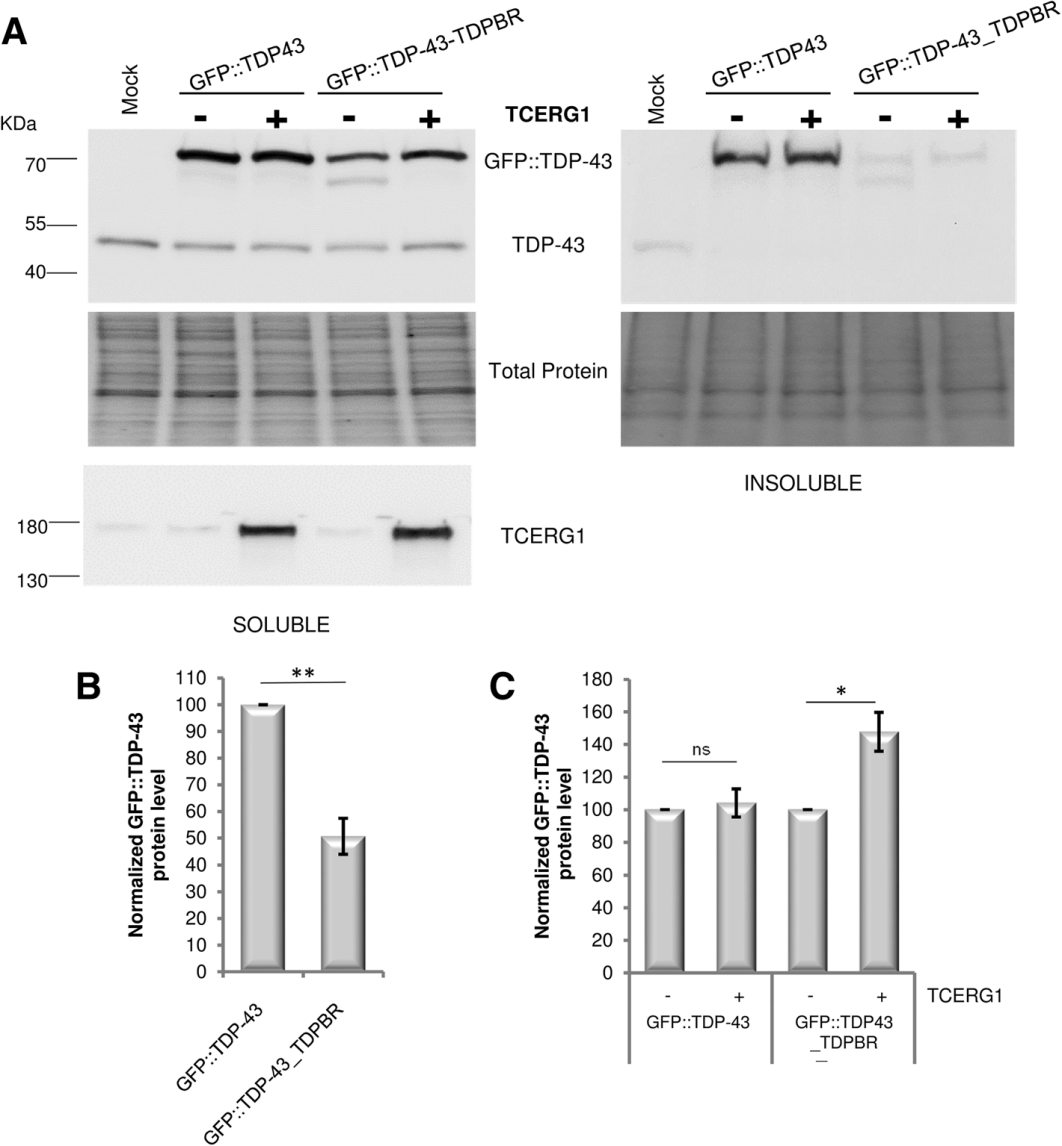
Human TCERG1 influences TDP-43 production in HEK293T cells. **a** Western blot analysis of HEK293T cells with different combinations of GFP::TDP-43 and TCERG1 expression plasmids. Expression of both proteins were detected using anti-TDP-43 or anti-TCERG1 antibodies. Representative result from four independent experiments is presented. Proteins were sequentially extracted in RIPA (soluble) and Urea (insoluble) buffers. Total protein was used as the loading control. **b, c** The normalized expression of the TDP-43 protein is reported in the graphs (mean ± SEM). Protein levels were compared between both genotypes by using Student’s t-test. ***: p < 0.001, *: *p* < 0.05

To validate these findings identified in *Drosophila* in mammalian cells, we first developed two hybrid constructs containing a *GFP::TDP-43* reporter gene fused (*GFP::TDP-43_TDPBR*) or not (*GFP::TDP-43*) to the *TDPBR* region, and compared their expression in HEK293 cells. As expected, introduction of the *TDPBR* sequence into the reporter construct resulted in a significant ~ 50% decrease (*p* = 0.0053) in the GFP::TDP-43 protein expression levels (Fig. 6a, b). Co-transfection of the *GFP::TDP-43_TDPBR* reporter construct with a construct encoding the human T7-tagged TCERG1 protein, resulted in a significant increase of TDP-43::GFP protein steady-state levels relative to control transfections (*p* = 0.0278) (Fig. 6a, c). In contrast, no significant increase in GFP::TDP-43 production was detected in the context of the *GFP::TDP-43* reporter construct (*p* = 0.6659). Together these results showed that the human TCERG1 protein can regulate TDP-43 protein production in mammalian cells, and that TCERG1-mediated regulation of TDP-43 production is also predominantly mediated by the *TDPBR* region.

## Discussion

TDP-43 is a critical RNA-binding factor that has been shown to play a central role in RNA metabolism. Cell functions and survival depend on the strict control of TDP-43 protein levels. TDP-43 expression is tightly regulated through an autoregulatory negative feedback loop mediated by the binding of TDP-43 protein in a specific region of its mRNA 3’UTR called *TDPBR* [5, 6, 8, 42, 54]. The *TDPBR* sensor region includes low-affinity binding sites for TDP-43 and the polyadenylation site pA1, the most efficient polyadenylation site of the *TDP-43* gene. In steady-state conditions, most TDP-43 production within cells comes from the transcript that uses the polyadenylation site pA1. Increase in TDP-43 nuclear levels results in an increased occupancy of the *TDPBR* that in turn suppresses usage of the pA1 site, resulting in elongation of transcripts beyond pA1. The elongated transcripts present an acceptor site for normally silent intron that contains the *TDPBR* region and the pA1 sequence. The exclusion of this intron forces the system to use suboptimal polyadenylation sites. The mRNAs using these alternative polyadenylation sites show an increased incidence of alternative splicing, and are partially retained in the nucleus and/or degraded.

To identify genetic modulators of TDP-43 production in vivo, we used an autoregulatory TDP-43 *Drosophila* model previously developed and characterized by our group [55]. This *Drosophila* transgenic model is based on the expression of the human *TDP-43* cDNA under the control of the *TDPBR* sensor region. This *TDP-43_TDPBR Drosophila* model recapitulates key features of the self-regulatory process of the steady-state levels of TDP-43 proteins described previously in mammalian and cellular models, namely alternative splicing events, differential usage of polyadenylation sites, nuclear retention of the transcripts, and a decrease in steady-state mRNA levels.

In this study, we report the identification of the *CG42724 Drosophila* gene as a genetic modulator of TDP-43 production in vivo. We showed that *CG42724* overexpression caused a drastic increase of TDP-43 protein steady-state levels, whereas *CG42724* down-regulation resulted in a decrease of TDP-43 accumulation. The study of the underlying molecular mechanisms allowed us to highlight that the CG42724 protein influences both qualitatively and quantitatively the *TDP-43_TDPBR* mRNA transcripts pattern. We found that CG42724 overexpression promotes the inclusion of the *TDPBR* sensor region as well as the production of transcripts ending at the pA1 polyadenylation, isoforms that can be efficiently released into the cytoplasm for protein translation. Of course, additional mechanisms could also be involved. Notably, we observed that TCERG1 expression resulted in a slight increase of *TDP-43* mRNA steady-state level, which could also contribute to the increase in TDP-43 protein accumulation. Importantly, this effect predominantly depends on the presence of the *TDPBR* region.

To date, very little is known about the *Drosophila* CG42724 protein. The protein is composed of 1123 residues and contains three WW domains at its N-terminus followed by six FF domains at its C-terminus. The CG42724 protein was detected in affinity-purified *Drosophila* spliceosome [31]. On the other hand, CG42724 was identified as an RS-domain containing protein in a genome-wide survey of RS domain proteins [13]. Interestingly, the RS-domains are frequently found in proteins involved in pre-mRNA splicing.

The *CG42724* gene encodes a homolog of the human *TCERG1 (Transcription elongation regulator 1)* gene. The CG42724 and TCERG1 proteins share 35% sequence identity and 48% sequence similarity. The highest homology is observed in the WW and FF domains. Importantly, our data suggest a significant degree of functional conservation between flies and mammals regarding the regulation of TDP-43 production. Indeed, we showed that similarly to CG42724, human TCERG1 overexpression also caused an increase of TDP-43 protein steady-state levels in mammalian cells.

TCERG1, previously named CA150, is a highly conserved human nuclear protein, localized at the interface of nuclear speckles and presumed nearby transcription sites [59, 60]. TCERG1 was originally identified as a component of an active cellular fraction that supported Tat activated transcription from the HIV-LTR [67, 68]. Consistent with a role in elongation [21], TCERG1 is found associated with elongation factors and RNA Polymerase II (RNAPII) holoenzyme [16, 30, 68]. Accumulating evidence also implicates TCERG1 in pre-RNA splicing regulation. TCERG1 interacts with splicing factors [30, 43] and has been identified in highly purified spliceosomes in multiple studies [25, 43, 45, 50, 57]. TCERG1 can affect pre-mRNA splicing of several splicing reporters [43, 48, 53, 60], and of putative cellular targets identified by microarray analysis following *TCERG1* knockdown [49, 53]. Based on these data, TCERG1 has been suggested to couple the transcribing RNAPII with spliceosome complexes to regulate co-transcriptional splicing events, a hypothesis that was supported by the demonstration that TCERG1 regulates the alternative splicing of the *Bclx* gene through the modulation the RNAPII transcription rate [48].

Interestingly, TCERG1 and TDP-43 proteins have been linked to common aspects of mRNA life cycle, namely transcription, pre-mRNA alternative splicing and polyadenylation site selection. Uncovering the mechanism of action of TCERG1 on TDP-43 production is complicated by their multifaceted functions, but also by the fact that RNAPII transcription, alternative splicing and alternative polyadenylation can be influenced reciprocally. All these processes are tightly coupled and coordinated.

We showed in this study that these processes of regulation predominantly depend on the presence of the *TDPBR* region. We demonstrated previously that the negative regulatory activity of the *TDPBR* region is specifically dependent on TDP-43 expression [55], suggesting that the TDP-43 protein itself could be implicated in the TCERG1-mediated regulation of TDP-43 production. One hypothesis would be that TCERG1 protein interferes with the binding of TDP-43 on the *TDPBR* region, possibly by competition through binding the same mRNA site. Indeed, even if TCERG1 has not been described as an RNA-binding protein, it has been shown that the protein associates in vitro with the *Bcl-x* pre-mRNA [48]. Alternatively, it could act by “sequestration” of TDP-43 out of the transcripts. However, to our knowledge, proteomic studies performed in several cell lines did not identify TCERG1 as a potential TDP-43 interacting partner [11, 27, 63, 75]. In contrast, they share several common interacting partners, such as the SRSF1, SRSF3, SRSF7, and SF3B splicing factors.

As a component of the splicing machinery, TCERG1 could also modulate the spliceosome assembly and activity. Consistent with this possibility, it has been shown that spliceosome assembly across the 3’UTR region induced by TDP-43 is a key event in the reduction of the amount of TDP-43 [8]. The 3’UTR intron 7 recognition by the splicing machinery somehow marks the bulk of the transcript for nuclear retention and degradation. Therefore, TCERG1 overexpression could alter the recognition of the intron 7 splicing sites, and consequently favor the recognition of pA1.

Otherwise, it has been shown that TDP-43 overexpression causes a rise in RNAPII density from the *TDPBR* sequence to the downstream region [5]. Such a pausing of RNAPII could influence polyA site usage [28] and the more efficient recognition of weaker splice sites [15, 40]. Thus, the pausing of RNAPII in the *TDPBR* region could interfere with the recognition of pA1, forcing the use of suboptimal polyadenylation sites. As mentioned above, TCERG1 modulates the rate of RNAPII transcription by increasing its elongation rate [21]. Therefore, in our experimental model, TCERG1 overexpression could release paused polymerase, and therefore allow the use of pA1 and the production of transcripts that can be transported into the cytoplasm for protein synthesis. It is also possible that TCERG1 works at the interface of RNAPII and the splicing machinery. Indeed, as mentioned above, TCERG1 can regulate alternative splicing events by modulating the rate of RNAPII transcription [48]. Thus, although our data are fully consistent with the known functions of the human protein TCERG1, they do not discriminate between several potential mechanisms. Of course, these mechanisms are non-exclusive, it is possible for all to work together.

Transcriptomic studies showed that TCERG1 is widely and highly expressed in the brain (cerebral cortex, hippocampus, lateral ventricle, and cerebellum). Interestingly, TCERG1 has already been implicated in the pathogenesis of the neurodegenerative disorder Huntington’s disease (HD). TCERG1 interacts with the huntingtin (HTT) protein and has been associated with the morphological deposits related to the disease [34]. TCERG1 could play a neuroprotective role in HD because its overexpression rescues neuronal cell death due to mutant HTT neurotoxicity [3]. TCERG1 has also been identified as a genetic modulator of Tau neurotoxicity in a genetic screen performed in our laboratory [10]. However, to date the molecular mechanisms underlying TCERG1-mediated neuronal effects remain largely unknown. A recent study showed that TCERG1 is required for normal neurite development in cultured cells, and suggested that abnormal regulation of the transcription and/or alternative splicing of TCERG1-specific targets may therefore play a role in the pathogenesis of TCERG1-associated neurological disorders [49].

## Conclusions

To conclude, using a *Drosophila* model that recapitulates key features of the TDP-43 auto-regulatory feedback loop, we have identified TCERG1 as a modulator of TDP-43 production in vivo*.* Further studies will be necessary to unravel the exact mechanisms through which TCERG1 modulates TDP-43 production. Nevertheless, regardless of underlying mechanisms, our data suggest the possibility that targeting TCERG1 could be therapeutic in TDP-43 proteinopathies.

## Additional file

Additional file 1: **Figure S1.** Schematic representation of the organization of the human *TDP-43* gene. (TIF 282 kb)

Additional file 2: The table lists the detailed genotypes of the flies used in this study. (DOC 64 kb)

Additional file 3: The table lists the primers used in this study. (TIF 573 kb)

Additional file 4: **Figure S2.** Quantification of *CG42724* steady-state mRNA levels by RT-QMPSF. (A) Schematic representation of the *CG42724* transcription unit, the relative location of RNAi target sites (green boxes) and the RT-QMPSF amplicon (red box). (B) Expression analyses of *CG42724* mRNA transcript by RT-QMPSF. The single-stranded cDNA was PCR-amplified using one pair of primers spanning *CG42724*, yielding a 173 bp product, and a pair of primers spanning the reference gene *Cyp1* (162 bp). The diagrams shown were obtained from *GMR > +* (control), *GMR > UY5237* flies. The y-axis displays fluorescence in arbitrary units, and the x-axis indicates the size in bp. The electropherogram of the *GMR > +* (blue) and *GMR > UY5237* (green) flies were superimposed by adjusting the peaks obtained for the control amplicon to the same level. (TIF 456 kb)

Additional file 5: **Figure S3.** Light micrographs of new-born *Drosophila* adult eyes. Compared to control flies (*GMR-Gal4* > +), expression of *CG42724* (*GMR-Gal4 > UY5237*) or *TDP-43_TDPBR* (*GMR-Gal4 > UAS-TDP-43_TDPBR*) alone triggered no structural defects. Similarly, flies co-expressing *CG42724* and *TDP-43_TDPBR* have no external phenotype. (TIF 25334 kb)

Additional file 6: **Figure S4.** Homology TCERG1 and CG42724. Alignment of the human TCERG1 and *Drosophila* CG42724 proteins. TCERG1 and CG42724 share 35% sequence identity and 48% sequence similarity. The highest homology is observed in the WW domain (blue) and the FF domain (red). Alignment was performed using DRSC Integrative Ortholog Prediction Tool. (TIF 338 kb)

Additional file 7: **Figure S5.** Quantification of *TDP-43* steady-state mRNA levels by RT-QMPSF. (A) Schematic representation of the *TDP-43* transcription unit and the relative location of the RT-QMPSF amplicons. (B) Expression analyses of *TDP-43* mRNA transcripts by RT-QMPSF. This assay is based on simultaneous PCR amplification of short fluorescent fragments in a single tube. The single-stranded cDNA was PCR-amplified using: TDP-43^F1/R1^ yielding a 115 bp product, TDP-43^F2/R2^ that yielded fragments of 132 bp and *CG42724* that produced an amplicon of 173 bp (Additional file 1: Figure S1A). *RpL13A* (141 bp) and *Cyp1* (162 bp) cDNAs were amplified as internal references. The number of cycles of amplification was determined by testing a range of cycle numbers in order to remain in the linear phase of the PCR. Fluorescent amplicons were separated on a genetic analyzer and the resulting fluorescent profiles were analyzed. The diagrams shown were obtained from *GMR > +* (control, blue), *GMR > TDP-43_TDPBR* (red) or *GMR > TDP-43_TDPBR, UY5237* (green) flies. The y-axis displays fluorescence in arbitrary units, and the x-axis indicates the size in bp. The electropherograms were superimposed by adjusting the peaks obtained for the control amplicons to the same level. (C) TDP-43 amplicons were amplified using a TDP-43-specific primer (F3) and an oligo-dT adapter primer (AUAP). The diagrams shown were obtained from *GMR > +* (control, blue), *GMR > TDP-43_TDPBR* (red) or *GMR > TDP-43_TDPBR, UY5237* (green) flies. *Cyp1* cDNAs was amplified as internal reference. The electropherograms were superimposed by adjusting the peaks obtained for the control amplicons to the same level. Note that the “mis-alignement » of the longest pics is due to the imprecise sizing of the fragment > 1 kb. (TIF 1151 kb)

Additional file 8: **Figure S6.**. Purity of subcellular fractions. Cytoplasmic/nuclear fractionation was performed on *GMR > +* (control), *GMR-Gal4 > UAS-TDP-43_TDPBR* or *GMR-Gal4 > UAS-TDP-43_TDPBR, UY5237* transgenic flies. Nuclear (N) and cytoplasmic (C) fractions were qualified by performing Western blot experiments. Results shown are representative of 3 independent biological replicates. β-tubulin was used as a cytosolic marker, while histone H3 was used as a nuclear marker. Total protein was used as the loading control by Stain-free technology. (TIF 683 kb)
