# Supplementary figures and images for: Identification of TCERG1 as a new genetic modulator of TDP-43 production in Drosophila
Source: Acta Neuropathol Commun. 2018 Dec 12;6:138. doi: 10.1186/s40478-018-0639-5 (PMC6292132; doi:10.1186/s40478-018-0639-5)

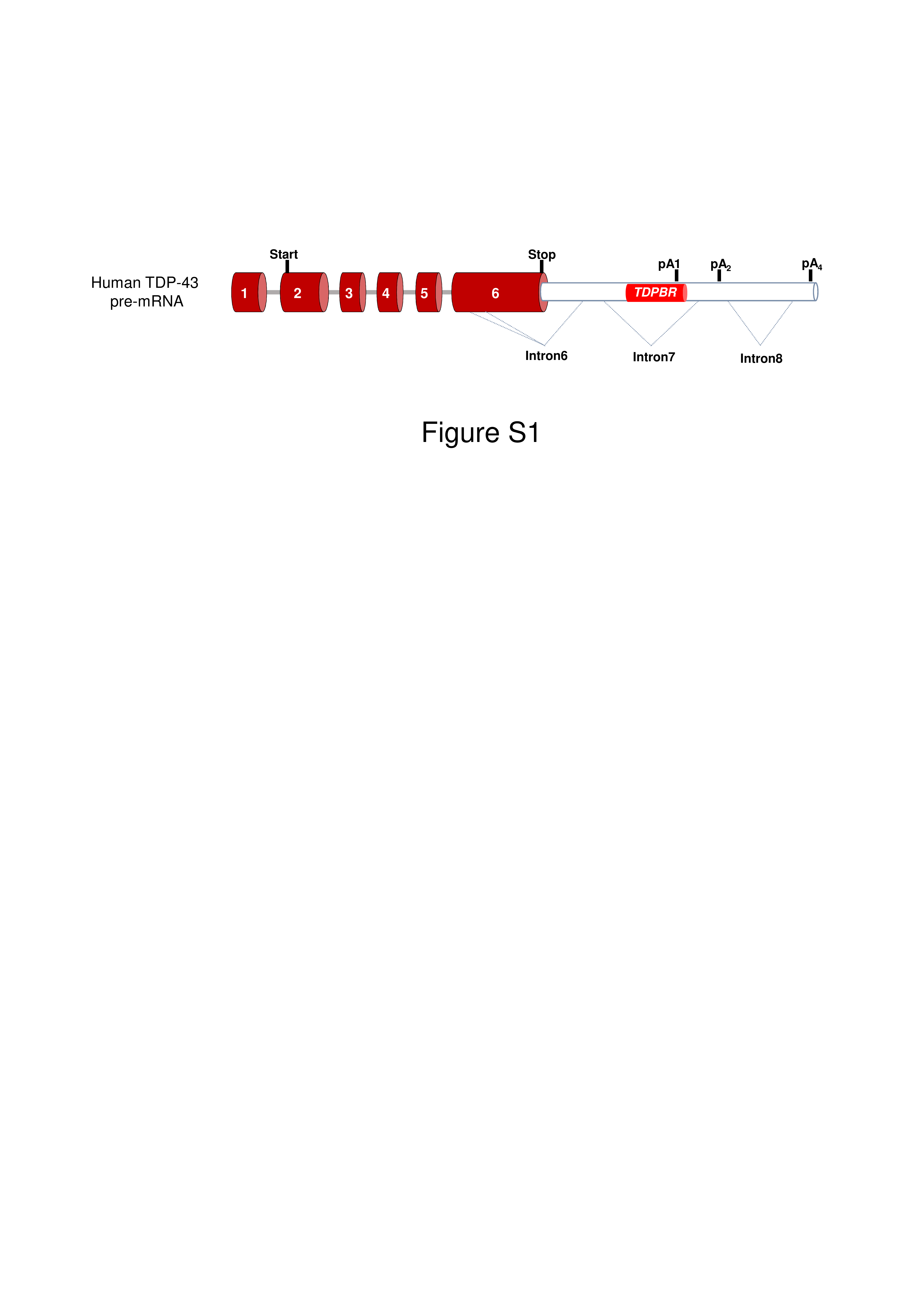

Supplement: Supplementary file 1 — Figure S1. Schematic representation of the organization of the human TDP-43 gene. (TIF 282 kb) [file 40478_2018_639_MOESM1_ESM.tif]

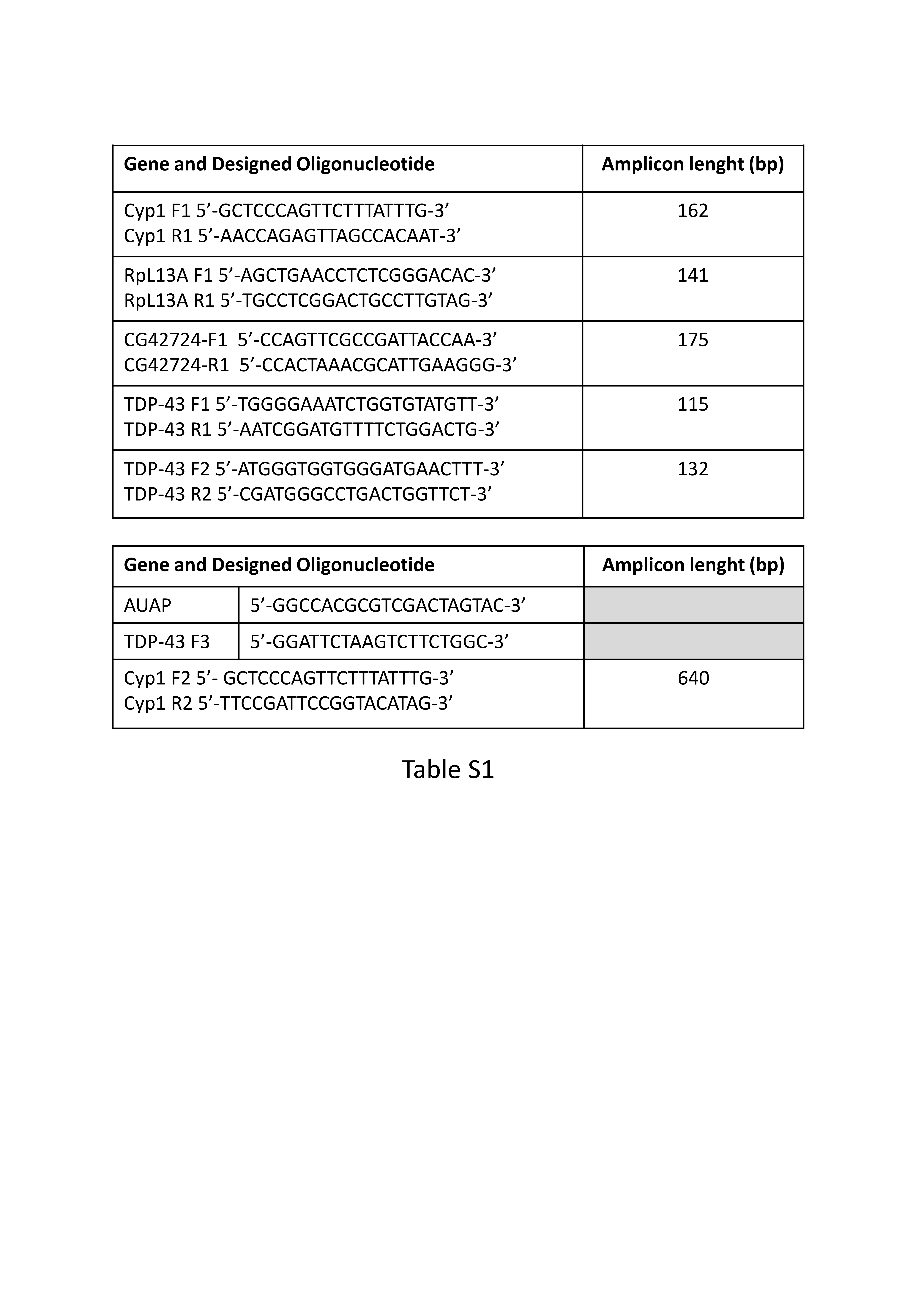

Supplement: Supplementary file 3 — The table lists the primers used in this study. (TIF 573 kb) [file 40478_2018_639_MOESM3_ESM.tif]

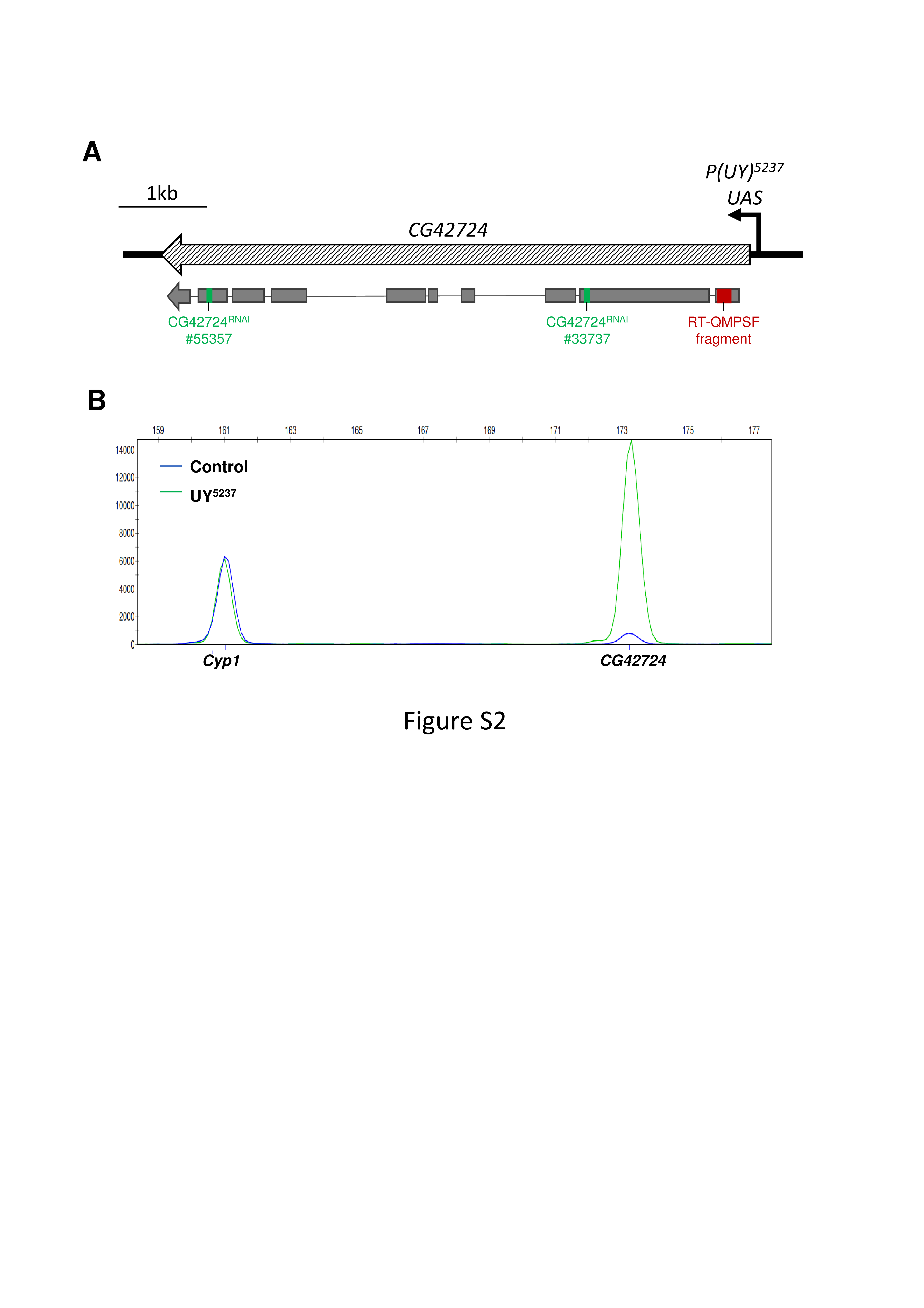

Supplement: Supplementary file 4 — Figure S2. Quantification of CG42724 steady-state mRNA levels by RT-QMPSF. (A) Schematic representation of the CG42724 transcription unit, the relative location of RNAi target sites (green boxes) and the RT-QMPSF amplicon (red box). (B) Expression analyses of CG42724 mRNA transcript by RT-QMPSF. The single-stranded cDNA was PCR-amplified using one pair of primers spanning CG42724, yielding a 173 bp product, and a pair of primers spanning the reference gene Cyp1 (162 bp). The diagrams shown were obtained from GMR > + (control), GMR > UY5237 flies. The y-axis displays fluorescence in arbitrary units, and the x-axis indicates the size in bp. The electropherogram of the GMR > + (blue) and GMR > UY5237 (green) flies were superimposed by adjusting the peaks obtained for the control amplicon to the same level. (TIF 456 kb) [file 40478_2018_639_MOESM4_ESM.tif]

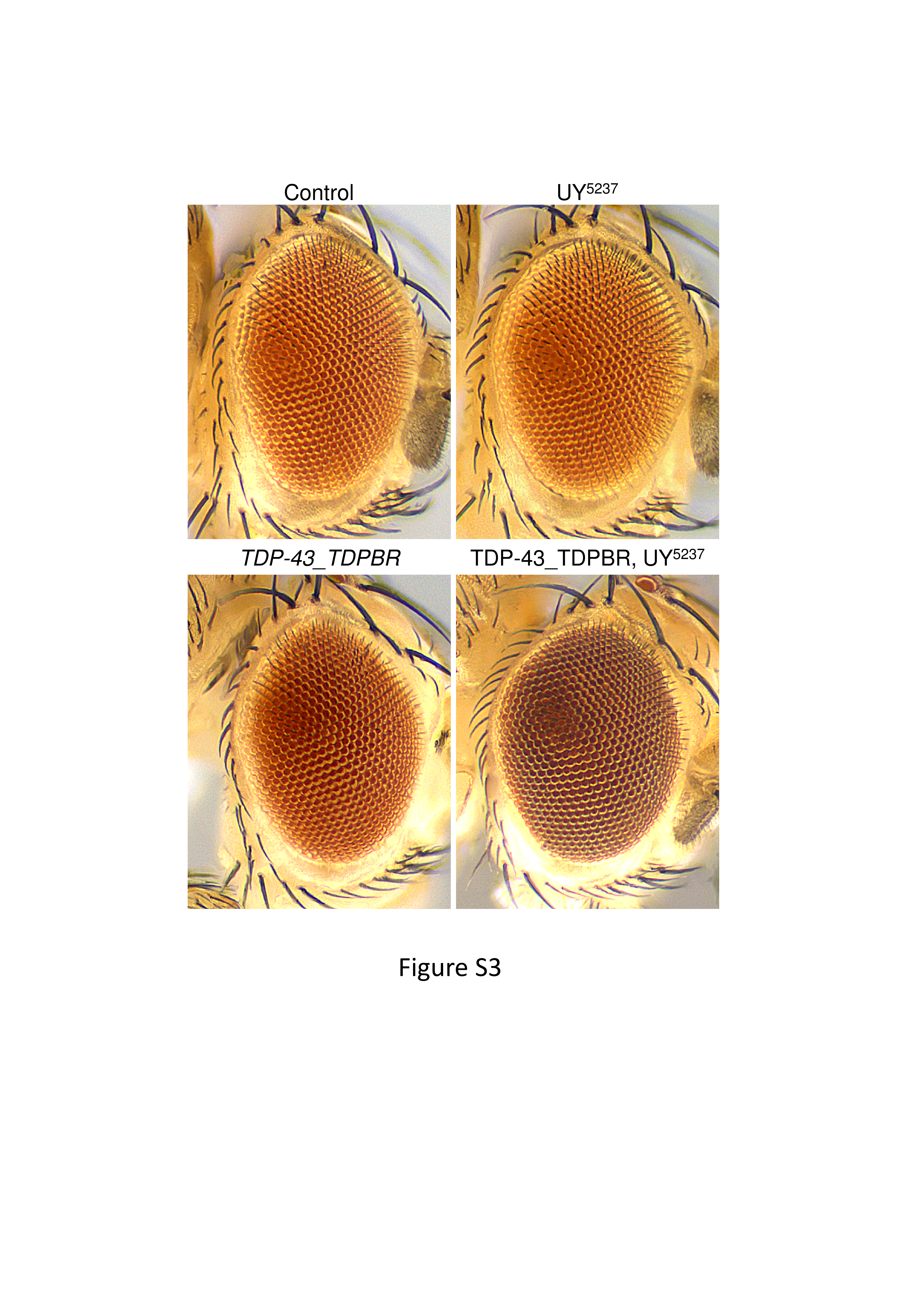

Supplement: Supplementary file 5 — Figure S3. Light micrographs of new-born Drosophila adult eyes. Compared to control flies (GMR-Gal4 > +), expression of CG42724 (GMR-Gal4 > UY5237) or TDP-43_TDPBR (GMR-Gal4 > UAS-TDP-43_TDPBR) alone triggered no structural defects. Similarly, flies co-expressing CG42724 and TDP-43_TDPBR have no external phenotype. (TIF 25334 kb) [file 40478_2018_639_MOESM5_ESM.tif]

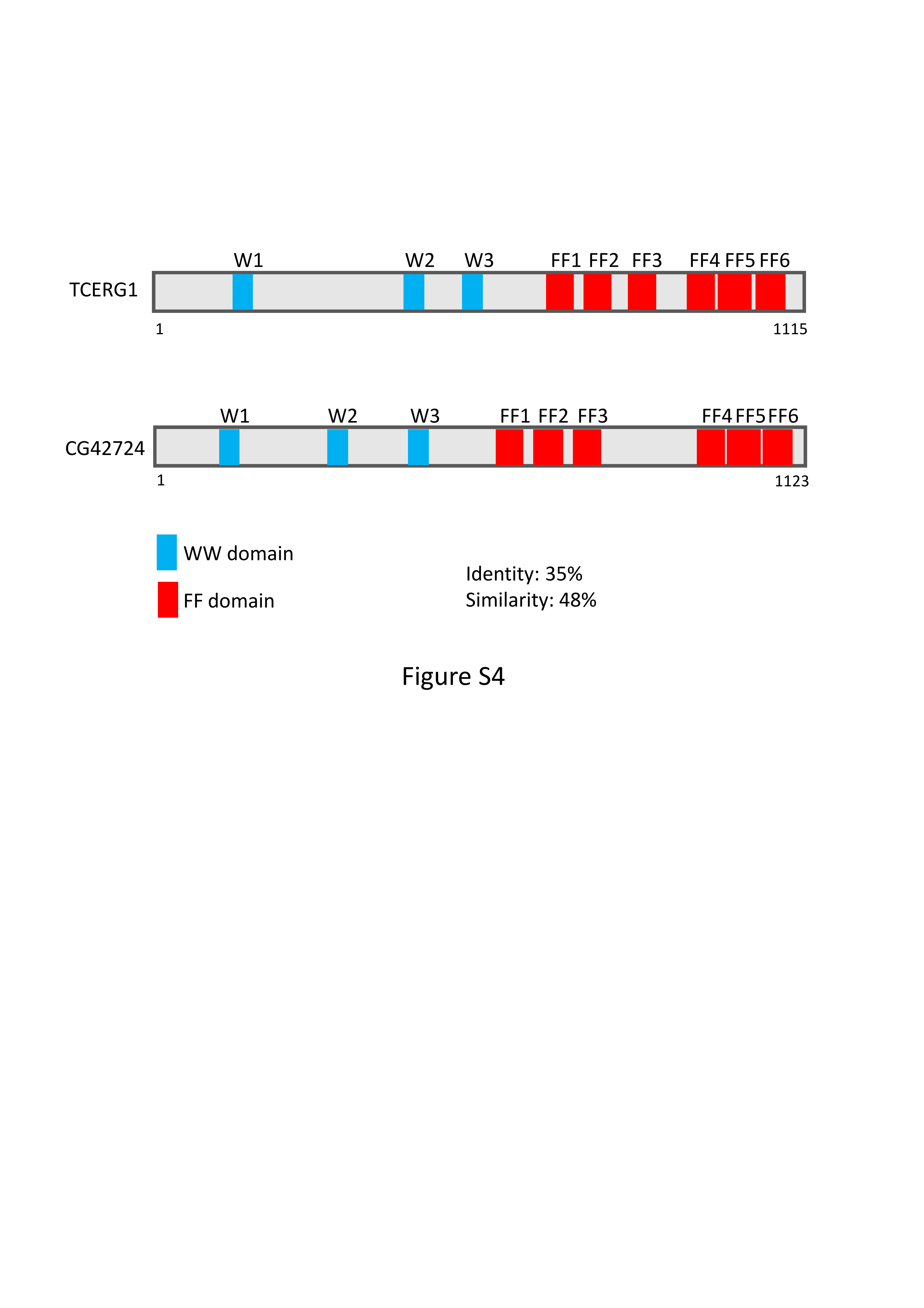

Supplement: Supplementary file 6 — Figure S4. Homology TCERG1 and CG42724. Alignment of the human TCERG1 and Drosophila CG42724 proteins. TCERG1 and CG42724 share 35% sequence identity and 48% sequence similarity. The highest homology is observed in the WW domain (blue) and the FF domain (red). Alignment was performed using DRSC Integrative Ortholog Prediction Tool. (TIF 338 kb) [file 40478_2018_639_MOESM6_ESM.tif]

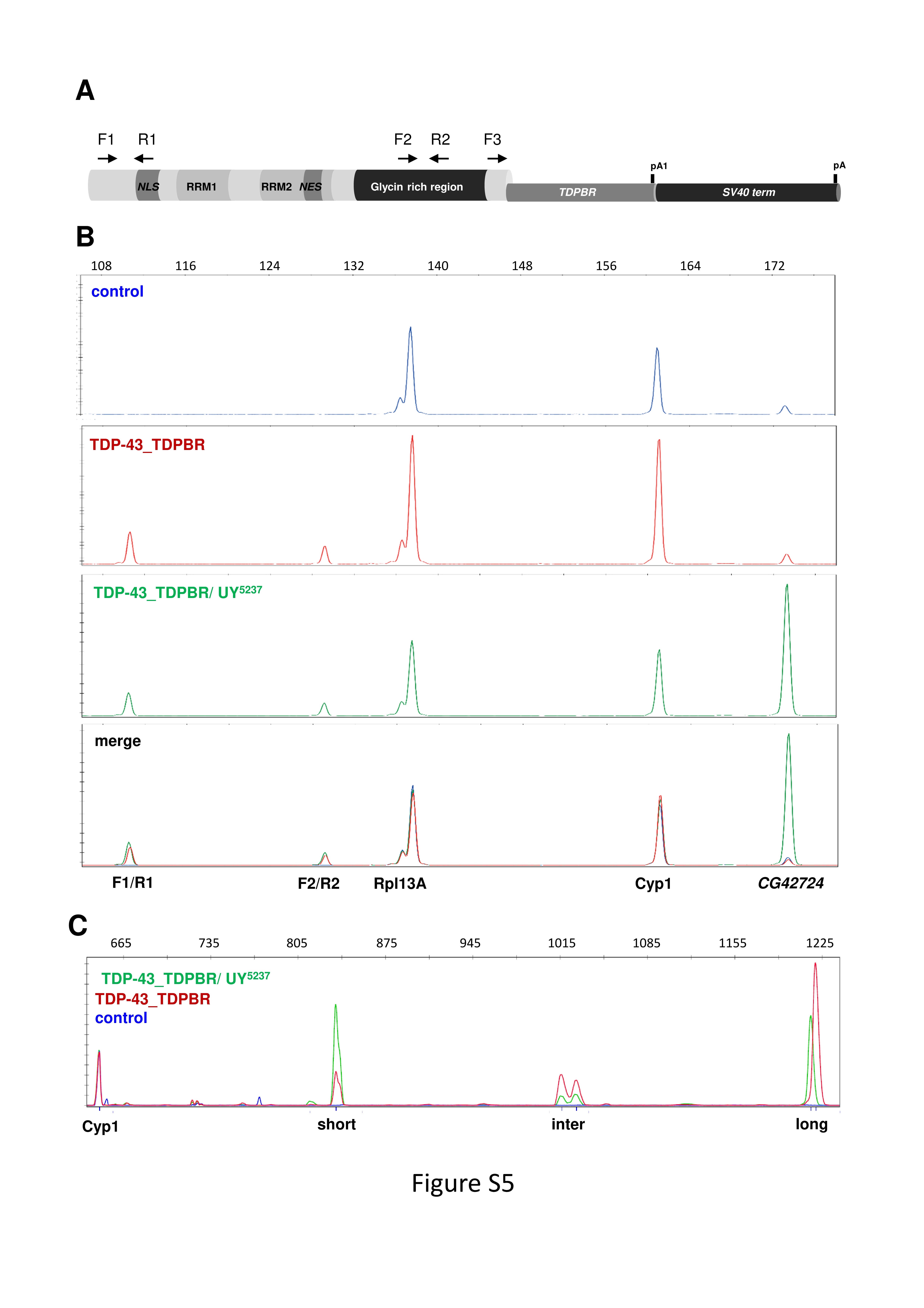

Supplement: Supplementary file 7 — Figure S5. Quantification of TDP-43 steady-state mRNA levels by RT-QMPSF. (A) Schematic representation of the TDP-43 transcription unit and the relative location of the RT-QMPSF amplicons. (B) Expression analyses of TDP-43 mRNA transcripts by RT-QMPSF. This assay is based on simultaneous PCR amplification of short fluorescent fragments in a single tube. The single-stranded cDNA was PCR-amplified using: TDP-43F1/R1 yielding a 115 bp product, TDP-43F2/R2 that yielded fragments of 132 bp and CG42724 that produced an amplicon of 173 bp (Additional file 1: Figure S1A). RpL13A (141 bp) and Cyp1 (162 bp) cDNAs were amplified as internal references. The number of cycles of amplification was determined by testing a range of cycle numbers in order to remain in the linear phase of the PCR. Fluorescent amplicons were separated on a genetic analyzer and the resulting fluorescent profiles were analyzed. The diagrams shown were obtained from GMR > + (control, blue), GMR > TDP-43_TDPBR (red) or GMR > TDP-43_TDPBR, UY5237 (green) flies. The y-axis displays fluorescence in arbitrary units, and the x-axis indicates the size in bp. The electropherograms were superimposed by adjusting the peaks obtained for the control amplicons to the same level. (C) TDP-43 amplicons were amplified using a TDP-43-specific primer (F3) and an oligo-dT adapter primer (AUAP). The diagrams shown were obtained from GMR > + (control, blue), GMR > TDP-43_TDPBR (red) or GMR > TDP-43_TDPBR, UY5237 (green) flies. Cyp1 cDNAs was amplified as internal reference. The electropherograms were superimposed by adjusting the peaks obtained for the control amplicons to the same level. Note that the “mis-alignement » of the longest pics is due to the imprecise sizing of the fragment > 1 kb. (TIF 1151 kb) [file 40478_2018_639_MOESM7_ESM.tif]

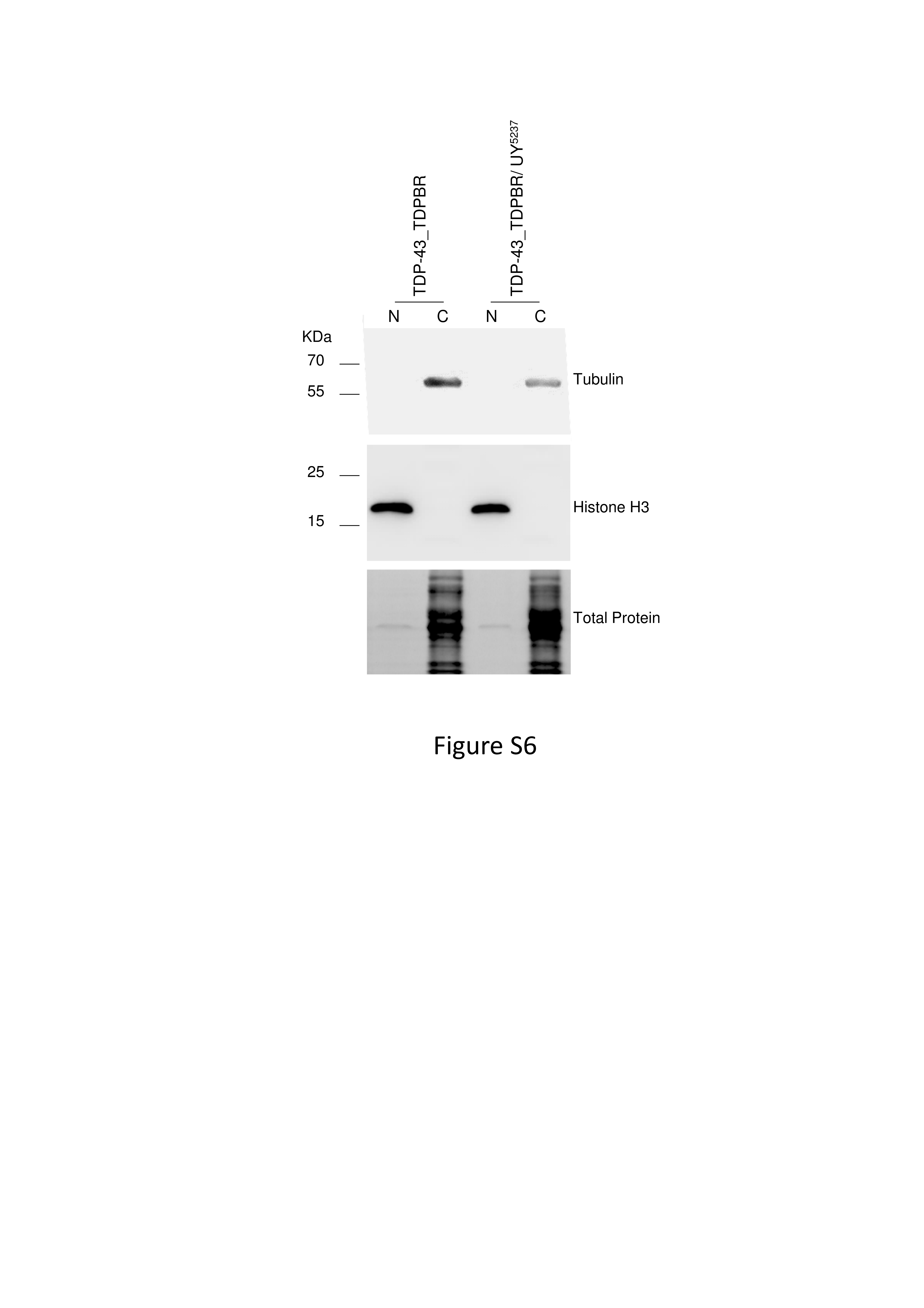

Supplement: Supplementary file 8 — Figure S6.. Purity of subcellular fractions. Cytoplasmic/nuclear fractionation was performed on GMR > + (control), GMR-Gal4 > UAS-TDP-43_TDPBR or GMR-Gal4 > UAS-TDP-43_TDPBR, UY5237 transgenic flies. Nuclear (N) and cytoplasmic (C) fractions were qualified by performing Western blot experiments. Results shown are representative of 3 independent biological replicates. β-tubulin was used as a cytosolic marker, while histone H3 was used as a nuclear marker. Total protein was used as the loading control by Stain-free technology. (TIF 683 kb) [file 40478_2018_639_MOESM8_ESM.tif]
