# Supplementary material for: Identification of TCERG1 as a new genetic modulator of TDP-43 production in Drosophila
Source: Acta Neuropathol Commun. 2018 Dec 12;6:138. doi: 10.1186/s40478-018-0639-5 (PMC6292132; doi:10.1186/s40478-018-0639-5)
Supplement: Supplementary file 2 — The table lists the detailed genotypes of the flies used in this study. (DOC 64 kb) [file 40478_2018_639_MOESM2_ESM.doc]

**Supplemental Materials and Methods**

**Detailed Fly Genotypes**

|  | **Detailed Fly genotypes** |
| --- | --- |
| **Figure 1** |  |
| *GMR > +* | *+/+ ; GMR-Gal4/+* |
| *GMR > TDP-43_TDPBR* | *+/+ ; GMR-Gal4 , UAS-TDP-43_TDPBR / +* |
| *GMR > TDP-43_TDPBR , UY5237* | *+/+ ; GMR-Gal4 , UAS-TDP-43_TDPBR/+ ; UY5237/+* |
| *GMR > lacZ* | *+/+ ; GMR-Gal4/UAS-lacZ* |
| *GMR > lacZ , UY5237* | *+/+; GMR-Gal4, UAS-lacZ/+; UY5237/+* |
| *GMR > FUS* | *+/+ ; GMR-Gal4/UAS-FUS::RFP* |
| *GMR > FUS , UY5237* | *+/+ ; GMR-Gal4, UAS-FUS::RFP/+ ; UY5237/+* |
| *GMR > TDP-43_TDPBR , CG42724^RNAi#55357^* | *+/+ ; GMR-Gal4,UAS-TDP-43_TDPBR/+;UAS- CG42724^RNAi#55357^/+* |
| *GMR > TDP-43_TDPBR , CG42724^RNAi#33737^* | *+/+ ; GMR-Gal4,UAS-TDP-43_TDPBR / UAS-CG42724^RNAi#33737^* |
| **Figure 2** |  |
| *GMR > +* | *+/+ ; GMR-Gal4/+* |
| *GMR > UY5237* | *+/+ ; GMR-Gal4/+ ; UY5237/+* |
| *GMR > CG42724^RNAi#55357^* | *+/+ ; GMR-Gal4/+ ; UAS-CG42724^RNAi#55357^/+* |
| *GMR > CG42724^RNAi#33737^* | *+/+ ; GMR-Gal4/UAS-CG42724^RNAi#33737^* |
| **Figure 3** |  |
| *GMR x2 > +* | *+/+ ; GMR-Gal4/ GMR-Gal4* |
| *GMR x2 > UY5237* | *+/+ ; GMR-Gal4; UY5237 / GMR-Gal4 ; +* |
| *GMR x2 > TDP-43_TDPBRx2* | *+/+ ; GMR-Gal4, UAS-TDP-43_TDPBR/ GMR-Gal4, UAS-TDP-43_TDPBR* |
| *GMR x2 > TDP-43_TDPBRx2 , UY5237* | *+/+ ; GMR-Gal4, UAS-TDP-43_TDPBR/ GMR-Gal4, UAS-TDP-43_TDPBR ; UY5237/+* |
| **Figure 4** |  |
| *GMR > TDP-43_TDPBR* | *+/+ ; GMR-Gal4, UAS-TDP-43_TDPBR/+* |
| *GMR > TDP-43_TDPBR ,UY5237* | *+/+ ; GMR-Gal4, UAS-TDP-43_TDPBR/+ ;UY5237/+* |
| *GMR > TDP-43* | *+/+ ; GMR-Gal4, UAS-TDP-43/+* |
| *GMR > TDP-43 , UY5237* | *+/+ ; GMR-Gal4, UAS-TDP-43/+ ; UY5237/+* |
| **Figure 5** |  |
| *GMR > TDP-43_TDPBR* | *+/+ ; GMR-Gal4, UAS-TDP-43_TDPBR/+* |
| *GMR > TDP-43_TDPBR , UY5237* | *+/+ ; GMR-Gal4, UAS-TDP-43_TDPBR/+ ; UY5237/+* |
| **Figure S2** |  |
| *GMR > +* | *+/+ ; GMR-Gal4/+* |
| *GMR > UY5237* | *+/+ ; GMR-Gal4/+ ; UY5237/+* |
| **Figure S3** |  |
| *GMR > +* | *+/+ ; GMR-Gal4/+* |
| *GMR > UY5237* | *+/+ ; GMR-Gal4/+ ; UY5237/+* |
| *GMR > TDP-43_TDPBR* | *+/+ ; GMR-Gal4, UAS-TDP-43_TDPBR/+* |
| *GMR > TDP-43_TDPBR ,UY5237* | *+/+ ; GMR-Gal4, UAS-TDP-43_TDPBR/+ ; UY5237/+* |
| **Figure S5** |  |
| *GMR > +* | *+/+ ; GMR-Gal4/+* |
| *GMR > TDP-43_TDPBR* | *+/+ ; GMR-Gal4, UAS-TDP-43_TDPBR/+* |
| *GMR > TDP-43_TDPBR ,UY5237* | *+/+ ; GMR-Gal4, UAS-TDP-43_TDPBR/+ ; UY5237/+* |
| **Figure S6** |  |
| *GMR > TDP-43_TDPBR* | *+/+ ; GMR-Gal4, UAS-TDP-43_TDPBR/+* |
| *GMR > TDP-43_TDPBR , UY5237* | *+/+ ; GMR-Gal4, UAS-TDP-43_TDPBR/+ ; UY5237/+* |

**Primary antibodies**

The following primary antibodies were used: rabbit polyclonal anti-Histone3 (1:5,000; Abcam, Paris, France), mouse monoclonal anti-β-tubulin E7 (1:5,000; Developmental Studies HybridomaBank).
